# Relevance of Targeting Oxidative Stress, Inflammatory, and Pro-Resolution Mechanisms in the Prevention and Management of Postoperative Atrial Fibrillation

**DOI:** 10.3390/antiox14040414

**Published:** 2025-03-29

**Authors:** Abir Attia, Kalai Mangai Muthukumarasamy, Doa’a G. F. Al-U’Datt, Roddy Hiram

**Affiliations:** 1 Montreal Heart Institute, Montreal, QC H1T 1C8, Canada; 2 Department of Pharmacology and Therapeutics, McGill University, Montreal, QC H3A 0G4, Canada; 3 Department of Biochemistry and Physiology, Faculty of Medicine, Jordan University of Science and Technology, Irbid 22110, Jordan; 4 Department of Medicine, Faculty of Medicine, University of Montreal, Montreal, QC H3T 1J4, Canada

**Keywords:** atrial fibrillation, inflammation, pro-resolution, catheter ablation, POAF

## Abstract

**Highlights:**

**What are the main findings?**

- Cardiac oxidative stress and inflammation are suspected to trigger arrhythmias after cardiac surgery.
- Unresolved inflammation leads to the development of atrial fibrosis, an irreversible substrate for AF.

**What are the implications of the main findings?**

- Resorption of atrial fibrosis might help prevent and cure POAF.
- New anti-inflammatory and antifibrosis strategies have emerged as potential therapies against POAF.

**Abstract:**

Atrial fibrillation (AF) is the most common cardiac arrhythmia. AF can lead to severe complications, including stroke, myocardial infarction, and sudden death. AF risk factors include pathological aging and conditions such as obesity, diabetes, and hypertension. Clinical data revealed that cardiothoracic and non-cardiothoracic surgeries are also important risk factors for AF. Post-operative AF (POAF) is associated with important public health costs caused by increased hospitalization, frequent emergency room visits, and enhanced healthcare utilization, which altogether lead to a low quality of life for the patients. Hence, POAF is a major clinical challenge, and there is an urgent need for the development of novel therapeutic strategies. Interestingly, evidence from clinical and fundamental research converges to identify cardiac oxidative stress and atrial inflammation as the common denominators of all AF risk factors. Unresolved inflammation is suspected to provoke cardiac fibrosis, which is an important contributor to cardiac arrhythmias and AF. Antioxidant, anti-inflammatory, and pro-resolution strategies may help to combat post-operative cardiac remodeling and POAF. This article aims to review the current scientific evidence supporting the role of inflammation in the pathogenesis of POAF and explore potential novel therapeutic strategies to prevent and mitigate inflammation in the management of AF.

## 1. Introduction

Atrial fibrillation (AF) stands as the most prevalent form of cardiac arrhythmia [1]. AF is characterized by irregular electrical activity occurring in the heart’s upper chambers, the atria [2]. AF can lead to the formation of blood clots, stroke, heart failure, and sudden death [3]. The management of AF varies based on multiple factors, such as age, symptom severity, frequency of symptoms, and the presence of comorbidities [4]. The main goals in treating AF include heart rate regulation, prompt restoration of normal heart rhythm, and the mitigation of stroke risk [5]. Pharmaceutical approaches are essential in AF management, as they also contribute to managing heart rate, reinstating normal heart rhythm, and averting blood clot formation [6]. Examples of these medications encompass beta-blockers, calcium (Ca^2+^) channel blockers, and anti-coagulation drugs [4,5,6,7,8]. Furthermore, electrophysiological radiofrequency catheter ablation (RFCA) stands out as a catheter-based procedure that does not require surgery and is designed to isolate and potentially eradicate abnormal electrical foci accountable for AF [9].

Ageing is the main AF risk factor, as 70% of AF patients are 65 and older [10]. AF risk factors also include obesity, diabetes, hypertension, pulmonary hypertension, and cardiomyopathy [11,12,13]. It is well established that a particular group of AF patients are subjected to post-operative AF (POAF) [14]. Cardio-thoracic interventions are considered as important risk factor for POAF with an incidence of 20–40% [15,16]. POAF is common after both cardiothoracic and non-cardiothoracic surgeries [16,17]. The incidence of AF following non-cardiac surgeries ranges from 3% to 30%, with a notably higher occurrence following thoracic procedures [18]. POAF is a common complication after valve surgery, with incidence rates varying between 37% and 50% [19]. Specifically, patients undergoing aortic valve replacement in conjunction with coronary artery bypass grafting (CABG) exhibit a 49% incidence of POAF [20]. Conversely, the occurrence of new-onset AF is lower following trans-catheter aortic valve replacement (TAVR), with an incidence of 9.9%, and similarly lower following post-transplantation, with an incidence of approximately 10.1% [21,22].

Inflammation has been proposed as a potential mechanism underlying the development of the arrhythmogenic substrate to AF [23], particularly in the context of POAF following cardiac surgery, where the timing of AF onset coincides with the peak activation of the inflammatory response [24]. Moreover, RFCA for atrial arrhythmias has been linked to elevated levels of inflammatory markers, such as C-reactive protein (CRP), indicative of inflammation and myocardial injury, which may contribute to an increased risk of early thrombotic events following AF ablation [25]. Although the pathophysiology of POAF is complex, evidence suggests that POAF patients are characterized by a specific inflammatory profile marked by the overexpression of circulating cytokines, including interleukin-(IL)-6 [26].

The mechanisms underlying the pathophysiology of AF remain incompletely elucidated. Hence, the management of POAF is complex. New-onset POAF that reverts to sinus rhythm prior to hospitalization discharge following cardiac surgery necessitates intensive monitoring, as these patients are at high risk for POAF recurrence and stroke [15].

This narrative review discusses the evidence of pro-inflammatory and pro-fibrosis signals in POAF and the relevance of anti-inflammatory approaches in managing this condition. In addition, we speculate on the impact of pro-resolution strategies, recognizing the growing evidence on the role of specialized pro-resolution mediators (SPMs) in promoting the termination of chronic inflammation and potentially preventing cardiac fibrosis [27,28].

## 2. Incidence of Postoperative Arrhythmias After Cardiac Surgeries

The emergence of AF as a postoperative complication following surgical interventions prompts an investigation into the complex relationships between these procedures and the development of AF. While the mechanistic underpinnings of this link remain intricate, this part of the review seeks to delve into the nuanced relationship between surgical procedures and the occurrence of AF, focusing on high-impact cardiac surgeries, thoracic interventions, and congenital cardiac procedures.

AF frequently occurs following noncardiac thoracic surgeries such as lobectomy, pneumonectomy, and esophagectomy [29]. It manifests in approximately 10% to 20% of cases [30]. Numerous studies have highlighted the well-documented association between cardiac surgeries and AF.

Noteworthy research by Helgadottir et al. [31] and Mathew et al. [32] explores mechanistic pathways and potential risk factors contributing to the heightened incidence of AF following cardiac surgeries, including CABG. The incidence of AF following isolated CABG ranges from 20 to 30%, while isolated valve surgeries have a higher incidence, around 35 to 40%. Both combined have a higher incidence of POAF, which has been reported in various studies from 35% to greater than 60% [33,34]. Kohno et al. demonstrated that AF recurrence was increased within six months following a first-time isolated aortic valve replacement [35]. Furthermore, chronic inflammation has been associated with persistent AF after mitral valve surgery [36]. Clinical studies have shown that patients undergoing surgical aortic replacement (SAVR) or TAVR to treat severe aortic stenosis are vulnerable to POAF [37,38]. A meta-analysis evaluated the risk of POAF after SAVR compared to TAVR in patients from seven randomized clinical studies, including PARTNER1/2/3 [39,40,41], CoreValve [42], SURTAVI [43], Notion [44], and Evolut [45]. It has also been shown that patients receiving SAVR (3935 subjects) were significantly more susceptible to POAF (33.3%) than those undergoing TAVR (9.7%; 3999 patients) [45,46].

Atrial arrhythmias pose the most common complication for adults diagnosed with congenital heart disease (CHD), often leading to heightened morbidity rates and frequent hospital admissions [47]. While intra-atrial reentrant tachycardias (IART) are typically the predominant type of arrhythmia observed initially, the incidence of AF rises with advancing age, surpassing IART rates among individuals aged 50 and older [48]. Notably, AF tends to manifest at an earlier age in CHD patients, presenting a significantly elevated risk compared to their counterparts without CHD [49].

Patients undergoing myocardial revascularization surgery (MRS) experience a reduced occurrence of postoperative AF, typically ranging from 30% to 40%, in contrast to those undergoing valve surgery, where the incidence is around 60% [50]. The utilization of extracorporeal circulation (ECC) is also linked to a higher likelihood of AF, although some studies suggest that there is no significant difference in incidence between cases where ECC is used and cases where it is not [50].

In the management of end-stage heart failure, heart transplantation (HTx) remains the gold standard treatment [51]. Cardiac arrhythmias are among the major early complications observed among HTx patients [52,53]. In a study involving 639 HTx patients, it was shown that 2.3% had no history of AF before HTx but developed AF after HTx. In addition, 34.3% HTx patients had AF before the cardiac transplantation and did not develop POAF after HTx. Interestingly, 11.4% of these HTx patients had AF before and after HTx [54].

These data suggest that although new-onset AF is non-negligible after HTx, pre-existing substrate and vulnerability to AF are important contributors of POAF post-HTx. (Table 1).

## 3. Paradox of Catheter Ablation in the Management of POAF

Ectopic foci linked to the onset of AF are often identified near the pulmonary vein ostia within the left atrium (LA) [55]. Precise localization of such sites of archaic electrical automaticity enables targeted and optimizing ablation interventions [56]. Radiofrequency catheter ablation (RFCA) is based on pulmonary vein (PV) isolation (PVI) and involves cauterizing the atrial myocardium around the PV to electrically isolate them and re-establish the normal propagation of the electrical influx in the atria [57]. Ultimately, RFCA is a crucial strategy for rhythm control in the treatment of paroxysmal AF that does not respond to medication [56,57]. RFCA is among the most frequently performed cardiac ablation procedures globally [58]. Ablation therapy is considered for patients with refractory AF who remain symptomatic or those who are unable to tolerate drug therapy. This consideration is especially applicable to younger individuals without structural heart disease, as well as those aged 65 and older [10]. Catheter ablation is a beneficial treatment for individuals experiencing symptomatic AF, aiming to enhance their quality of life and minimize morbidity. Nonetheless, the sustained success of AF catheter ablation over the long term is not yet optimal, as AF recurrence rates have been reported to be between 20% and 50% at one year [59].

With significant advancements in ablation technologies, a randomized, single-blinded trial comparing radiofrequency ablation and cryoballoon ablation demonstrated a reduction in AF burden of greater than 98% [60]. However, the rate of first recurrence of AF remained substantial, with a one-year efficacy rate of 53% [60]. The first 3 months following ablation are critical. Approximately 25% of patients seek emergency care, with 10% requiring hospitalization within 30 days after AF ablation [61,62].

The underlying mechanisms of early transient AF following ablation remain unclear. Possible explanations include a temporary stimulating effect of radiofrequency (RF) energy due to the inflammatory response post thermal injury and/or pericarditis; a transient disruption in the autonomic nervous system balance, potentially serving as an arrhythmia trigger; and a delayed impact of RFCA, possibly due to the growth or maturation of ablation lesions in the days following the procedure [63]. Another possible explanation for these early recurrences of AF is that they may be associated with post-RFCA inflammation, edema, and the healing process [64]. Early recurrence of atrial arrhythmia within the initial month post-ablation is likely influenced by transient factors such as inflammation, temporary autonomic imbalances, and the time required for lesion formation. Conversely, recurrence occurring after the first month is more likely indicative of ablation failure and pulmonary vein reconnection [65,66].

Hence, while ablation is an effective technique for addressing AF, and while it might be an option to treat POAF, it is still associated with a recurrence of AF in some cases. There is a risk that the procedure may not permanently eliminate AF, and the condition may recur shortly after the procedure or several months later.

## 4. Electrophysiological Mechanisms of POAF

The understanding of the electrophysiological mechanisms underlying the development of POAF is complex and remains incompletely elucidated. However, the knowledge is constantly progressing. Clinical and preclinical studies have helped to identify some of the major contributing factors that may provoke the development of the arrhythmogenic substrate for POAF.

In POAF, it has been suggested that the substrate for AF is developed due to (i) alterations of the atria induced by the surgery (myocardial insult) and (ii) preexisting cardiac abnormalities (pathological aging, arrhythmogenic cardiac condition), which are both responsible for increased vulnerability to AF post-surgery [67].

Among preexisting cardiac abnormalities predisposing to POAF, evidence has suggested that genetic factors play a major role [68]. Genetic factors contribute to the risk of POAF, with various variants influencing the development of the arrhythmogenic substrate after surgery [69]. In a genome-wide association study (GWAS) utilizing data from the UK Biobank, two variants, rs17042171 and rs17042081, located near the PITX2 gene, were significantly associated with POAF [69]. The study analyzed 144,196 surgical samples from patients who had undergone various surgical procedures [69]. The -174G/C variant in the promoter region of the interleukin-6 (IL-6) gene has been identified as implicated in the modulation of the inflammatory response to surgery, potentially influencing the development of POAF [70]. In a smaller cohort, the R87Q and P307S polymorphisms in the hKv1.5 gene were identified as potential contributors to POAF [71]. The G protein-coupled receptor kinase 5 (GRK5) gene was significantly associated with POAF in patients undergoing CABG, irrespective of perioperative β-blocker treatment [72]. Additionally, genetic polymorphisms in the intronic region of the lymphocyte antigen 96 (LY96) gene were linked to a decreased risk of POAF in patients undergoing CABG surgery [73].

Surgeries, including cardiac, thoracic, and non-cardiac surgeries, are often associated with cardiac alteration, including atrial stretching [17]. Atrial dilation affects the cardiomyocytes’ (CMs) structure and function by decreasing the expression and increasing the lateralization of gap junctions, including connexins (Cx) 40 and 43 [74]. Altered Cx40 and Cx43 are associated with slowed atrial conduction and increased vulnerability to AF [75]. Studies have shown that low magnesium levels after cardiac surgery are an indication for the onset of POAF [76]. Such hypomagnesemia is often observed in hospitalized patients who have received surgery [77]. In addition, it has been reported that patients with serum potassium (K+) concentrations below 4.5 mmol/L are 1.43 times more likely to develop POAF [78]. These data suggest that electrolyte disturbance during surgery might increase the risk of POAF. In this context, ion channel malfunction has been described as a major substrate for POAF [67,79]. Channelopathies involving inward K^+^-channels and decreased Na^+^-currents were described as potential contributors to POAF [75,80]. During cardiac surgery, alteration of CMs can be associated with decreased SERCA2a activity, perturbed calcium (Ca^2+^)-handling machinery, and malfunctional L-type Ca^2+^-channels, a phenomenon which has been described as highly arrhythmogenic in POAF [75,79,80]. Altered ion channels and gap-junction uncoupling provoke atrial conduction slowing, prolong effective refractory period (ERP), and prolong action potential duration (APD) [74].

**Figure 1:**
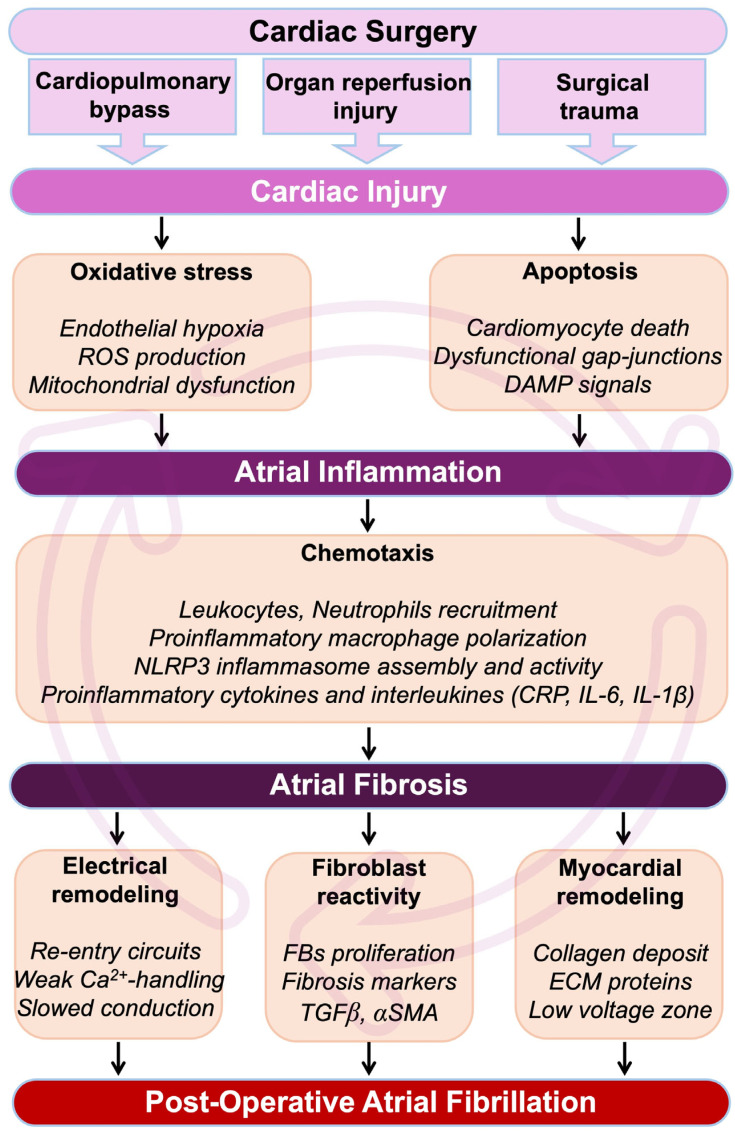
Pathophysiological cascade from cardiac surgery to POAF. During and after a cardiac intervention, the heart undergoes myocardial insults, which result in an immediate development of oxidative stress. Cardiac cells, including cardiomyocytes, will be affected by apoptosis, leading to the formation and circulation of damage-associated molecular pattern (DAMP) signals. Oxidative stress-induced cardiac hypoxia and the production of DAMP signals are major initiators of inflammation. Cardiac inflammation is characterized by the secretion of pro-inflammatory cytokines, interleukins, and cells in the myocardial environment. If unresolved, inflammation can become chronic, leading to the development of cardiac fibrosis and loss of function. In the atria, cardiac fibrosis leads to perturbation of the conduction, abnormal rhythm, re-entrant circuits, and refractoriness. Altogether, these events constitute a fertile arrhythmogenic substrate associated with an increased risk of AF. Abbreviations from Figure 1. α-SMA: alpha-smooth muscle actin; Ca^2+^: calcium; CRP: C-reactive protein; DAMP: damage-associated molecular pattern; ECM: extracellular matrix; FBs: fibroblasts; IL1β: interleukin 1β; IL6: interleukin 6; ROS: reactive oxygen species; NLRP3: NOD-like receptor family, pyrin domain containing 3; TGFβ: transforming growth factor beta.

In addition, evidence has suggested that hyperactivity of the sympathetic nerve system after surgery is an important contributor to POAF [81]. Altogether, these atrial electrical conduction abnormalities contribute to re-entry, automaticity, and increased vulnerability for the development and maintenance of POAF [67] (Figure 1).

## 5. Impact of Inflammation in POAF Pathophysiology

Inflammation is a biological response triggered by cardiac injury or infection. It aims to repair the damaged tissue and restore normal physiological functions [82]. If left unresolved, inflammation can trigger excessive production of cytokines and reactive oxygen species, potentially resulting in various disease states, including AF [83]. Inflammatory processes contribute to mechanisms like fibrosis, cellular apoptosis, and hypertrophy, which increase the susceptibility to AF when they occur within the atria [28]. Furthermore, reduced blood flow promotes microinjury or dysfunction of the endothelium in the atrial endocardium, facilitating the migration of immune cells into the atrial tissue [84].

In the pathogenesis of AF, it is hypothesized that both inflammation and the remodeling of the left atrium contribute significantly [85]. Indeed, atrial structural remodeling is a fundamental and important aspect in the development and progression of AF, with fibrosis in the left atrium being a key contributor to the manifestation of the condition [86]. Atrial fibrosis develops when there is an accumulation of collagen into the atrial extracellular matrix (ECM), leading to impaired atrial contraction [87]. In other words, perturbed atrial contractility results from an imbalance between fibrosis deposits and breakdown of the ECM components within the cardiac tissue [87]. Studies indicate a direct correlation between the degree of fibrosis and the persistence of AF [88].

**Figure 2:**
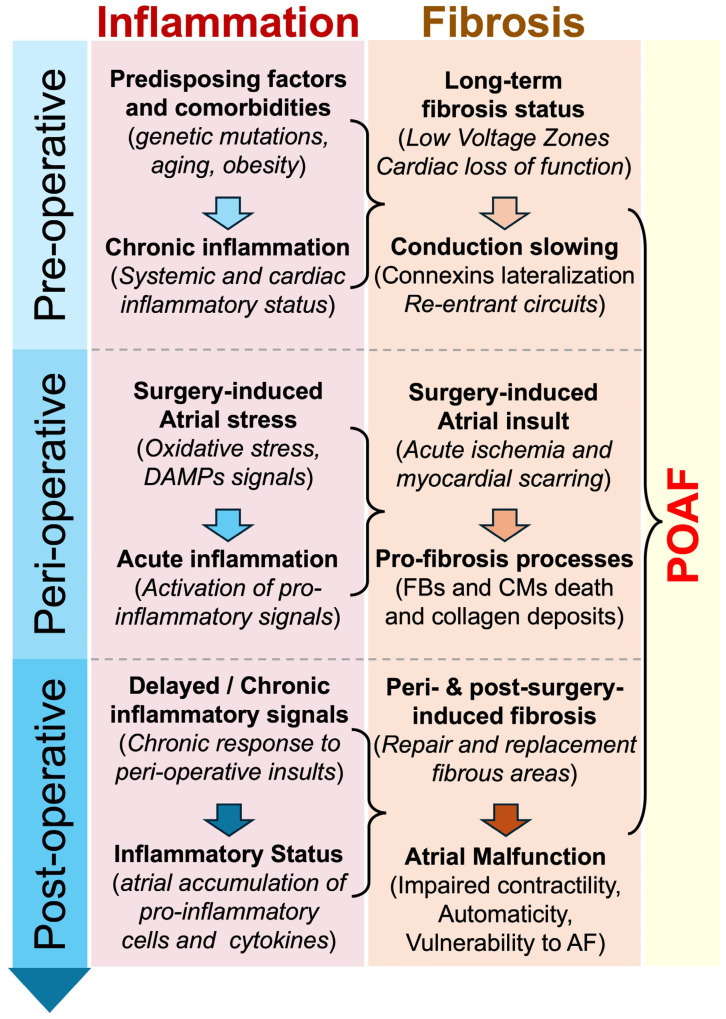
Proposed chronological implication of inflammation and fibrosis in POAF. Inflammation and fibrosis are suspected to play an important role in the development of POAF. Predisposing factors, peri-operative myocardial stress, or/and post-operative myocardial remodeling might be accompanied by the activation and maintenance of pro-inflammatory and pro-fibrosis signals, which participate in the development of arrhythmogenesis and POAF. Abbreviations from Figure 2. AF: atrial fibrillation; CMs: cardiomyocytes; DAMPs: damage-associated molecular patterns; FBs: fibroblasts; POAF: postoperative atrial fibrillation.

Patients with lone AF, occurring in otherwise healthy adults without underlying heart disease, have been observed to exhibit higher levels of collagen deposition compared to individuals with sinus rhythm [89]. This increased collagen deposition is also observed in patients with AF secondary to mitral valve disease, as opposed to those in sinus rhythm [90]. The inflammatory response triggered by cardiac surgery predominantly arises from the trauma inflicted during the operation, which includes the surgical procedure itself, the use of cardiopulmonary bypass (CPB), and the injury caused by organ reperfusion [91]. Patients undergoing these open-heart surgeries often also have pre-existing chronic inflammatory conditions, including atherosclerosis, myocardial infarction, and AF [92] (Figure 1 and Figure 2).

Surgical trauma leads to oxidative stress and the production of proinflammatory molecules and reactive oxygen species generation [93]. Various studies have revealed a correlation between systemic inflammation, oxidative stress, and the onset of POAF [94]. In POAF, there is a growing body of evidence suggesting that acute inflammation related to surgery plays a significant role in the development of pathogenesis [95]. Furthermore, patients with elevated postoperative leukocyte counts are notably more prone to developing POAF [96,97], and patients developing POAF tend to have a greater degree of monocyte activation [98]. Additionally, the heightened pre- and post-operative neutrophil/lymphocyte ratio in patients undergoing CABG may also be linked to a higher occurrence of POAF [99].

Previous studies using animal models have shown that activated neutrophils, upon adhering to cardiac myocytes, can induce alterations in myocyte electrical activity, which may contribute to arrhythmogenesis [100,101]. Another proinflammatory component of interest that has been shown to play an essential role in the development of AF is the NACHT, LRR, and PYD domain-containing protein 3 (NLRP3) inflammasome [102]. The activity of the NLRP3 inflammasome has been observed to increase in the atria of patients with both paroxysmal and long-standing persistent AF [102] (Figure 1).

## 6. Classical Anti-Arrhythmogenic Approaches in POAF Management

The clinical management of POAF is complex because it requires a rigorous approach to treat AF in the context of the specific surgery received by the patient [103]. The hemodynamic status is crucial to adopting the appropriate treatment for POAF [104]. In patients with hemodynamic instability, rhythm control strategies aiming for prompt restoration of sinus rhythm and cardioversion are required [105]. In patients with hemodynamic stability, rate and rhythm control are solicited [105]. Medications such as atrioventricular nodal blockers (calcium channel blockers, beta-blockers, digoxin) can be used to promote rate control [106].

In the context of POAF, the use of beta-blockers and calcium channel blockers must be administrated cautiously [107]. Catheter ablation is commonly used to promote rhythm control in AF, but this approach is complex in patients with POAF because catheter-based procedures and surgical interventions are often contraindicated due to prohibitive risk of complications [108].

According to guidelines from the American College of Cardiology/American Heart Association/Heart Rhythm Society and the European Society of Cardiology (ESC), the use of long-term oral anticoagulation is convenient after POAF complicating non-cardiac surgery (Class of Recommendation IIa, Level of Evidence B) and cardiac surgery (Class of Recommendation IIb, Level of Evidence B), given informed patient preferences [109,110].

## 7. Potential of Antioxidant, Anti-Inflammatory, and Pro-Resolution Strategies in POAF Management

The presence of a pro-inflammatory and pro-fibrosis environment before, during, or after cardiac surgery or ablation is believed to play a role in the emergence of POAF or early recurrence of AF post-ablation [111,112] (Figure 2). Consequently, numerous researchers have investigated the effectiveness of anti-inflammatory agents in averting POAF induced by inflammation [111,113]. Therefore, anti-inflammatory drugs may reduce the rate of AF incidence in post-surgery conditions [114]. Although mounting evidence suggests that inflammation plays a central role in the arrhythmogenesis of POAF [115,116], more investigations are required to consolidate the understanding of the beneficial mechanisms and effects of anti-inflammatory treatments. In this context, in June 2023, colchicine was the first anti-inflammatory medication approved by the US Food and Drug Administration (FDA) for the prevention of cardiac and cardiovascular events in adult patients with atherosclerotic cardiovascular disease (ASCVD) [117].

### 7.1. Colchicine

Recent years have witnessed a growing body of evidence highlighting the significant role of inflammation in the development of cardiovascular conditions. Colchicine, known for its potent anti-inflammatory properties, acts by inhibiting microtubule growth at low doses and supporting microtubule depolymerization at higher doses [118]. This disruption of microtubule proteins by colchicine inhibits the activity of the NLRP3 inflammasome, leading to a decrease in the secretion of pro-inflammatory cytokines and the formation of neutrophil extracellular traps (NETs) [119]. Consequently, colchicine has emerged as a promising therapeutic option for managing cardiovascular diseases [120]. It is approved for treating and preventing acute gout, as well as other inflammatory conditions like pericarditis [121]. Postsurgical inflammation, characterized by elevated levels of inflammatory biomarkers like C-reactive protein (CRP) and interleukin-6 (IL-6), is among the numerous potential contributors to POAF [122]. In recent studies, colchicine has demonstrated efficacy in lowering the incidence of POAF and early recurrence of AF following PVI. Additionally, it has been observed to decrease the levels of proinflammatory biomarkers such as CRP and IL-6 [123,124]. Administering colchicine at a dosage of 0.5 mg twice daily for a period of 90 days resulted in a reduction of both early and late recurrence of AF following catheter radiofrequency ablation [100,124]. Studies have also shown that initiating colchicine therapy perioperatively may lower the occurrence of postoperative AF [125]. In June 2023, low-dose colchicine 0.5 mg, with the branded name LODOCO, was approved by the FDA as the first anti-inflammatory atheroprotective cardiovascular treatment [126,127]. FDA approval of low-dose colchicine treatment aims to reduce the risk of MI, stroke, coronary revascularization, and cardiovascular death in adult patients with ASCVD or with multiple risk factors for CVD [117]. However, although the knowledge is constantly advancing about the mechanisms through which colchicine exerts its beneficial anti-inflammatory effects against cardiovascular diseases, the elucidation of the precise underlying biological processes involved in its potential role against POAF remains a high-interest area of investigation [115,116].

### 7.2. Immunosuppressant Agents

IL-6 plays a critical role in cardiovascular diseases, including AF [128,129]. Observational studies have reported a positive correlation between IL-6 levels and the incidence of AF [128,129]. In a recent study, Li et al. showed that the selective blockade of IL-6 trans-signaling by sgp130Fc on transverse aortic constriction (TAC)-challenged mice prevented AF inducibility [130]. The study demonstrated that the prevention of AF resulted from the improvement in slow conduction and conduction heterogeneity, which were induced by structural changes in the atria, including dilation and fibrosis, in addition to the reduction in connexin 40 along with the redistributed connexin 43 [130]. The implication of IL-6 in the new onset of AF amid COVID-19 raises interest in exploring IL-6 receptor antagonists, like tocilizumab, as potential preventive measures [131]. Notably, a meta-analysis conducted by the World Health Organization Rapid Evidence Appraisal for COVID Therapies (REACT) Working Group revealed that IL-6 receptor antagonists reduce all-cause mortality in COVID-19 patients compared to standard care or placebo [132]. Although this analysis did not directly demonstrate the preventive efficacy of IL-6 receptor antagonists against AF, the observed mortality benefits may indirectly suggest a potential link to AF prevention [132]. Since AF is a recognized risk factor for mortality in COVID-19 patients, preventing its occurrence could improve survival rates [131]. Additionally, a multicenter cohort study found a lower incidence of AF in COVID-19 patients treated with tocilizumab compared to those who did not receive the treatment. This implies that tocilizumab administration might reduce atrial fibrillation risk, potentially contributing to the observed mortality benefits [133]. A study by Yao et al. investigated the causal link between the NLRP3 inflammasome and AF [102]. This study demonstrated that increased activity of the NLRP3 inflammasome in atrial cardiomyocytes activates caspase-1 (Casp1), which subsequently stimulates the conversion of pro-inflammatory cytokines, such as pro-IL-1β and pro-IL-18, into their biologically active forms, thereby promoting AF. This development of AF was attenuated by genetic inhibition of NLRP3 [102]. Studies have demonstrated that resolvin D1 (RvD1) reduces the co-localization of NLRP3 with its subunits [134]. Additionally, RvD1 inhibits Casp-1 activation and IL-1β production [134,135].

### 7.3. Non-Steroid Anti-Inflammatory Drugs (NSAIDs)

Non-selective nonsteroidal anti-inflammatory drugs (NSAIDs), which inhibit both cyclooxygenase (COX)-1 and COX-2 as well as selective COX-2 inhibitors, are commonly prescribed to reduce inflammation and fever and alleviate pain [136]. These drugs primarily exert their effects by inhibiting COX enzymes, which are involved in the production of pro-inflammatory mediators [137]. NSAIDs have been demonstrated to be effective for pain management in CABG patients. Notably, patients treated with diclofenac required less morphine compared to the control group [138]. Administering NSAIDs during the early postoperative period following CABG surgery has been shown to be relatively safe and effective in reducing the occurrence of AF. Additionally, these medications may have a beneficial impact on shortening the length of hospital stay for these patients [139]. A network meta-analysis encompassing 85 trials that evaluated the occurrence of new-onset POAF suggested that the use of NSAIDs and statins may reduce the risk of POAF when compared to a placebo [140]. However, contrasting findings suggest that NSAID use may also increase the risk of AF. A population-based case-control study utilizing a prescription database demonstrated that the use of non-aspirin NSAIDs was associated with a heightened risk of developing AF or atrial flutter [141]. Additionally, a mean follow-up of 12.9 years from the Rotterdam Study, which evaluated NSAID use in elderly individuals, found that both current and recent use of NSAIDs were linked to a higher risk of AF compared to individuals who had never used these medications [142]. In a study using the United Kingdom primary care database, it was concluded that both steroidal anti-inflammatory drugs (SAIDs) and NSAIDs were associated with persistent and permanent AF. As these medications are commonly prescribed for inflammatory conditions, this association suggests that chronic inflammation may be a contributing factor in the development of AF [143]. To avoid the bias that the condition treated by NSAIDs could be a risk factor for AF and not the use of NSAIDs per se, the studies have attempted to select patients with equivalent baseline conditions, treated with NSAIDs or not [141,142,143]. In terms of arrhythmogenic mechanisms, it has been suggested that use of NSAIDs might decrease renal function and provoke increased blood pressure, leading to left-ventricular remodeling and AF [142]. Moreover, NSAID-induced nephrotic malfunction might lead to abnormally elevated levels of circulating potassium, which might contribute to arrhythmogenesis [142].

### 7.4. Pro-Resolution Approaches

Studies have shown that unresolved inflammation is responsible for the progression and aggravation of AF [135]. Clinical reports have revealed that POAF patients are characterized by a particular inflammatory and fibrosis profile marked by the overexpression of circulating inflammatory biomarkers, including IL6 and CRP [113]. Cardiac surgeries might be accompanied by myocardial insults, leading to the development of atrial inflammation and fibrosis [144]. On the other hand, evidence suggests that pro-resolution strategies are efficient in preventing postoperative pain and curing myocardial inflammation [145,146].

The concept of “resolution” describes the active endogenous mechanisms involved in the cessation of inflammation and mediated by specialized pro-resolution mediators, including D- and E-series resolvins (RvD1, RvE1) [147,148]. In animal models of myocardial infarction and fibrosis caused by perfusion-reperfusion and permanent ligation of the left anterior descending coronary artery, and a rat model of right heart disease provoked by pulmonary artery hypertension, RvD1 has been shown to decrease myocardial fibrosis and reduce the vulnerability to AF if administrated early enough, supposedly before the development of atrial fibrosis [82,135,149,150].

**Figure 3:**
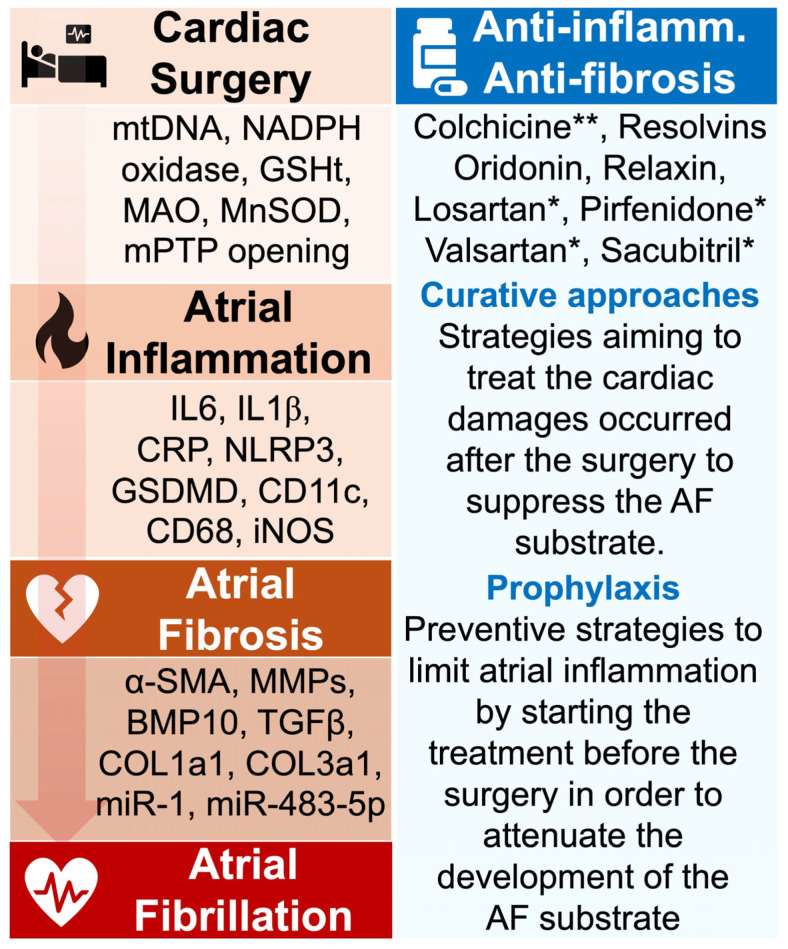
Aim of antioxidant, anti-inflammatory, and anti-fibrosis approaches in POAF management. The pathophysiological events from cardiac surgery to AF are marked by the secretion of active biomarkers that have been identified in clinical and experimental studies. Targeting such agents may contribute to preventing POAF. During cardiac surgery, the induced oxidative stress is characterized by the production of mtDNA, NADPH oxidase, and MnSOD, which have been associated with increased risk of AF. The atrial inflammation provoked by these signals is marked by the important secretion of proinflammatory molecules including IL6, CRP, and NLRP3 inflammasome. Persistence of the inflammatory status can generate the formation of atrial fibrosis, typically identified by elevated expression of α-SMA, TGF-β, and collagenase. Strategies aiming to prevent POAF have tested the impact of various anti-inflammatory medications, including colchicine and resolvins. Studies have revealed the ability of these drugs to attenuate myocardial inflammation and fibrosis, but more investigations are required to confirm their role in preventing AF. * FDA-approved medication; ** FDA-approved medication in the treatment of cardiac inflammation. Abbreviations from Figure 3. α-SMA: alpha-smooth muscle actin; BMP10: bone morphogenetic protein-10; CD11c: cluster of differentiation 11c; CD68: cluster of differentiation 68; COL1A1: collagen 1a1; COL3a1: collagen 3a1; CRP: C-reactive protein; GSDMD: gasdermin D; GSHt: glutathione transferase; IL1β: interleukin 1β; IL6: interleukin 6; iNOS: inducible nitric oxide synthase; MAO: monoamine oxidase; miR-1: microRNA-1; miR-483-5p: microRNA-483-5p; MMPs: matrix metalloproteinases; MnSOD: manganese-dependent superoxide dismutase; mtDNA: mitochondrial DNA; NADPH oxidase: nicotinamide adenine dinucleotide phosphate oxidase; NLRP3: NOD-like receptor family, pyrin domain containing 3; TGFβ: transforming growth factor beta.

These data suggest that preventive strategies involving pro-resolution treatment administrated in a prophylaxis manner could contribute to attenuating the risk of POAF. However, more experimental and clinical studies are required to evaluate the benefits of such approaches (Figure 3).

### 7.5. Anti-Fibrosis Strategies

As suggested above, fibrosis is an important event contributing to the initiation and aggravation of the arrhythmogenic substrate leading to POAF. Anti-fibrotic treatments are expected to promote beneficial effects in preventing or/and curing cardiac fibrosis in the context of POAF [151,152]. In a mouse model of myocardial infarction (MI), Künzel et al. described that mesalazine, an anti-inflammatory drug used to treat inflammatory bowel disease, could decrease perivascular myocardial collagen 1A1 (COL1A1) expression and perivascular fibrosis [151]. Furthermore, Silva et al. have shown that alamandine, a renin-angiotensin-aldosterone system (RAAS) modulator, could decrease collagen deposition, reduce cardiac hypertrophy, and attenuate oxidative stress by altering critical signaling pathways like the transforming growth factor beta (TGF-β) and 5′-Adenosine monophosphate-activated protein kinase (AMPK), contributing to preventing cardiac remodeling induced by pressure overload [152]. In 2020, Gao et al. highlighted the efficacy of oridonin, an NLRP3 inflammasome inhibitor, in reducing myocardial fibrosis, limiting infarct size, and preventing cardiac inflammation through the downregulation of pro-inflammatory cytokines (IL-1β and IL-18), positioning it as a promising therapeutic to treat acute MI-associated fibrosis [153]. Similarly, tetramisole, a tissue-nonspecific alkaline phosphatase (TNAP) inhibitor, was shown to improve cardiac function post-MI by mitigating fibrosis through AMPK-TGF-β and Smads signaling pathways [154]. Valsartan and sacubitril, which modulate PKG (protein kinase G) and Rho signaling, were shown to prevent cardiac fibrosis provoked by chronic pressure overload and heart failure with preserved ejection fraction (HFpEF) [155]. Hydrogen sulfide (H2S), which inhibits the Janus kinase signal transducer and activator of transcription (JAK/STAT) pathways, has been shown to be effective in reducing cardiac fibrosis in a rat model of diabetic cardiomyopathy [156]. In a rat model of cardiac hypertrophy, inhibition of the JAK/STAT pathway using parthenolide daily treatment was associated with decreased FBs-induced cardiac fibrosis and reduced CMs hypertrophy, suggesting important FBs–CMs interactions in arrhythmogenic cardiac remodeling [157]. The bone morphogenetic protein-7 (BMP-7) has been demonstrated to counteract the pro-fibrotic effects of TGF-β by attenuating cardiac hypertrophy and collagen deposition in patients and mice models of left-sided pressure overload [158].

In a rat model of aging, relaxin, an endogenous peptide hormone involved in vasodilation, has been shown to reverse age-related atrial fibrosis and persistent AF by reducing TGF-β1, decreasing collagen expression, and enhancing voltage-gated sodium channel function [159]. Losartan, an angiotensin receptor blocker, has shown similar effects by attenuating myocardial fibrosis and preventing cardiac hypertrophy in non-obstructive hypertrophic cardiomyopathy by suppressing TGF-β and collagen signaling pathways [160]. Likewise, in the MI context, it has been shown that FT011, an antifibrotic compound, reduces macrophage infiltration, decreases collagen deposition, limits interstitial fibrosis, and improves systolic function post-MI [161]. In addition, pirfenidone, a TGF-β1 inhibitor, has shown beneficial effects by reducing ventricular tachycardia rates, attenuating myocardial infarct size, and decreasing cardiac fibrosis area [162]. Collectively, these findings emphasize the importance of targeting cardiac fibrosis in the management of cardiac conditions associated with arrhythmogenesis and AF (Table 2).

Strategies aiming to modulate specific molecular pathways, such as TGF-β, NLRP3, AMPK, JAK/STAT, or/and transglutaminase 2 (TG2) have the potential to mitigate the pathological remodeling underlying the development of the arrhythmogenic substrate for atrial arrhythmias including AF [163].

### 7.6. Posterior Left Pericardiotomy

Clinical evidence suggests that cardiac surgery is commonly accompanied by pericardial effusion, which contributes to arrhythmogenesis and vulnerability to POAF [164]. Recent investigation revealed that posterior left pericardiotomy consisting of the drainage of pericardial effusion by posterior pericardial incision reduces the incidence of POAF [165].

These data from the PALACS (Effect of Posterior Pericardiotomy on the Incidence of Atrial Fibrillation After Cardiac Surgery) randomized controlled trial (NCT02875405), were obtained from 420 patients either treated or not treated with posterior left pericardiotomy [165]. In an observational study involving 2535 patients subjected to CABG and AVR, posterior pericardial chest tube drainage was associated with a significant reduction in POAF [166].

More investigations are required to improve and confirm the safety and beneficial impact of posterior left pericardiotomy in the prevention of POAF.

## 8. Discussion and Limitations

In this narrative review, we aimed to understand the impact of inflammation on the development of cardiac arrhythmias, including POAF, after cardiothoracic and non-cardiothoracic surgeries [17]. Mounting evidence suggests that circulating and myocardial inflammation play a crucial role in triggering the arrhythmogenic substrate responsible for the development and maintenance of POAF [167]. Such inflammatory signals may occur (i) before the surgery when the patient is exposed to predisposing factors and comorbidities; (ii) during the surgical procedure, mainly when the atria are affected during the intervention; or (iii) after the surgery when cardiac remodeling occurs as a consequence of the procedure [77,91,168] (Figure 2).

**Figure 4:**
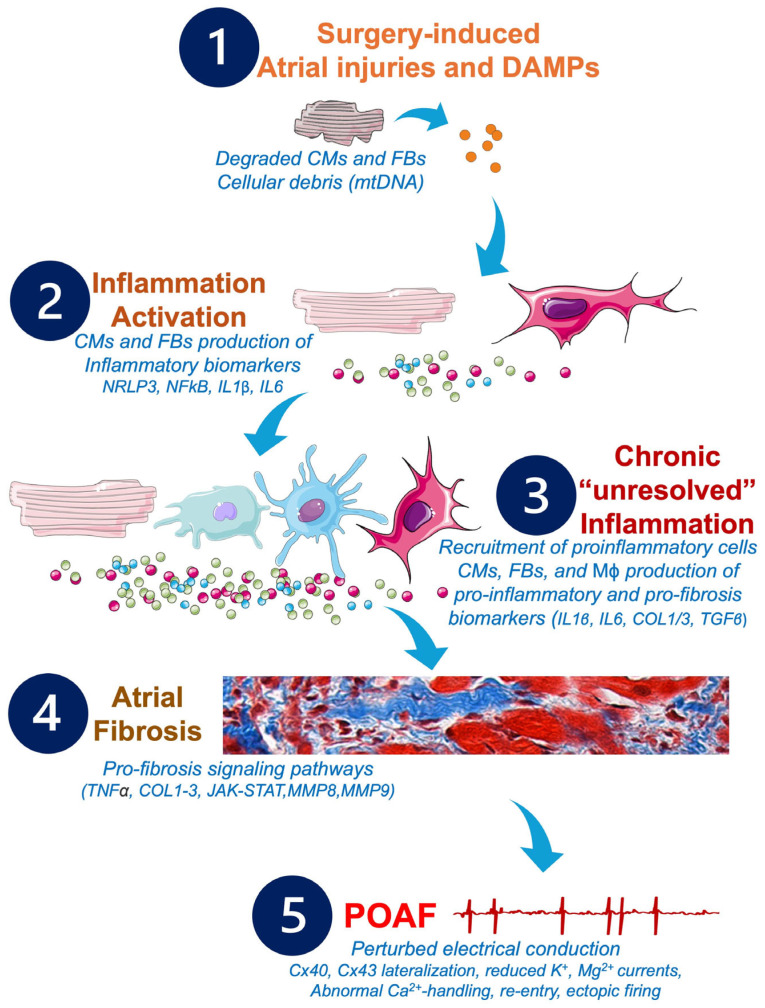
Potential pathophysiological cascade and molecular pathways to be targeted by future anti-inflammatory and anti-fibrosis treatments in POAF management. Cardiac and non-cardiac surgeries may provoke alterations of the CMs and FBs, which activate the inflammatory response, leading to production of further inflammatory biomarkers responsible for the remodeling of the atrial tissue, the development of atrial fibrosis, and the installation of POAF. Abbreviations from Figure 4. Ca^2+^: calcium; CMs: cardiomyocytes; COL1: collagen 1; COL3: collagen 3; Cx: connexin; DAMPs: damage-associated molecular patterns; FBs: fibroblasts; IL1β: interleukin 1β; IL6: interleukin 6; JAK/STAT: Janus kinase signal transducer and activator of transcription; K^+^: potassium; Mϕ: macrophages; Mg^2+^: magnesium; MMPs: matrix metalloproteinases; MnSOD: manganese-dependent superoxide dismutase; mtDNA: mitochondrial DNA; NFkB: nuclear factor kappa B; NLRP3: NOD-like receptor family, pyrin domain containing 3; TGFβ: transforming growth factor beta; TNFα: tumor necrosis factor alpha.

In response to insults, infections, or genetic abnormalities, the activation of inflammation affects the diverse cell types of the heart, including the endothelial cells, the macrophages, the FBs, and the CMs [169]. Various inflammatory pathways have been identified as being potentially involved in the pathophysiology of post-surgery arrhythmogenicity [170] (Figure 2). As described above, in the macrophage, FBs, and CMs, the activation of the NLRP3 inflammasome has been shown to promote POAF [171]. Inhibition of the JAK/STAT pathway has been shown to decrease FBs proliferation and CMs hypertrophy in response to pressure overload, and such phenomena are associated with the development of AF [172] (Figure 4).

The constantly growing knowledge about the major role of inflammation in triggering cardiac arrhythmias suggests that anti-inflammatory strategies must be taken into consideration in the management of heart rhythm disorders and POAF [173]. The precise kinetic of inflammation must be clarified to better prevent inflammation-associated arrhythmogenicity [174]. In this context, the choice of therapeutic strategy is complex and is between prophylactic or curative approaches [104,175].

For curative approaches, the POAF patients would receive anti-inflammatory treatment, starting peri- or post-surgery, in combination with the classic rate and rhythm control treatment, as a complementary medication to treat POAF [174,175,176].

For prophylactic approaches, the patient might be treated with anti-inflammatory medications in the long term, starting prior to the surgery and being maintained during and after the surgery to prevent inflammation induced by predisposing conditions and/or induced by the surgical procedure itself [177].

Among the prophylactic anti-inflammatory approaches, colchicine, an empiric medication known for its efficacy in treating gout, has recently been FDA-approved as the first anti-inflammatory medication for the prevention of CVD complications in the management of cardiac disorders [178].

Other anti-inflammatory approaches are mainly in the preclinical trial phase and need additional proof of efficacy. As described above, these approaches might involve corticoids, statins, NSAIDs, pro-resolution bioactive compounds, or antifibrosis strategies. In contrast, it is very important to notice that utilization of these molecules must be performed with caution because they are often accompanied by side effects [179]. In addition, some clinical studies have reported no effects of anti-inflammatory approaches in the prevention of POAF [180]. Overall, the treatment of POAF must be undertaken with caution with the rigorous decision of the physician.

## 9. Conclusions

During cardiothoracic and non-cardiothoracic surgeries, the intervention is likely associated with phenomena that cause cardiac remodeling, leading to the activation of inflammatory reactions. In addition, predisposing factors and comorbidities might contribute to the initiation of the inflammatory status before the surgery, which may be exacerbated and aggravated during and after the surgical intervention. If untreated, the inflammatory response generates cardiac fibrosis, which creates a physical barrier hindering the normal conduction of electrical current in the heart, particularly in the atria. This impaired conduction puts the atria at risk of POAF and other rhythm disorders. Hence, innovative therapeutic approaches should involve the complex but crucial management of inflammation to prevent and treat POAF.

## 10. Clinical Implications

POAF is a major concern affecting the management and quality of life of patients concerned. A better understanding of the pathophysiology of POAF may help to improve clinical practice and develop new therapeutic strategies. Such innovative approaches involve the utilization of promising anti-inflammatory medications that have shown or have the potential to show beneficial effects in experimental models and clinical trials.

## Figures and Tables

**Table 1:** Summary of studies analyzing arrhythmia incidence after cardiac surgery.

Reference	Surgery Type	Study Sample	Outcome Studied	Risk Group	AF Incident Rate
Jessurun et al. (2000) [36]	Mitral valve surgery with or without concomitant tricuspid surgery	162 patients	Long-term arrhythmia	Aged subjects, right ventricular pressure, tricuspid valve repair	AF persisted in 85% of patients with preoperative persistent AF
Mathew et al. (2004) [32]	CABG	1503 patients	New-onset atrial fibrillation	Advanced age, history of AF, COPD, valve surgery, postoperative withdrawal of a beta-blocker/ACE inhibitors/potassium supplementation/NSAIDs	32.3%
Onaitis et al. (2010) [30]	Lung cancer surgery—lobectomy	13,906 patients	POAF	Aged, male, increasing extent of operation, non-black race, stage 2 or greater tumors	12.6%
Helgadottir et al. (2012) [31]	CABG, OPCAB, AVR	744 patients	POAF	Aged, female, lower ejection fraction, higher EuroSCORE, less likely with history of smoking, more likely to have CHF.	44%
Kohno et al. (2017) [35]	First-time isolated AVR	157 patients	New-onset AF	Advanced age (>70 years) and absence of a postoperative β-blocker	36.9%
Mandalenakis et al. (2018) [49]	Operative repair in CHD patients	21 982 CHD patients, 219 816 matched control subjects	AF	Patients with atrial septal defect	21.99 times higher in CHD patients vs ctr; Surgical correction done in 40% of CHD patients had 3.56 times higher risk of AF
Altaii et al. (2020) [46]	TAVR	3999 patients	POAF	Lower risk of POAF after TAVR compared to SAVR	9.7%
Altaii et al. (2020) [46]	SAVR	3935 patients	POAF	Symptomatic severe aortic stenosis	33.3%
Darche et al. (2021) [54]	HTx	639 subjects	AF before and after HTx	Advanced donor age and extended ischemic duration	2.3%—No AF before but had AF after HTx 34.3%—AF before and not after HTx 11.4%—Had AF before and after HTx

Abbreviations from Table 1. ACE: angiotensin-converting enzyme; AF: atrial fibrillation; AVR: aortic valve replacement; CABG: coronary artery bypass grafting; CHD: congenital heart disease. CHF: chronic heart failure; COPD: chronic obstructive pulmonary disease; CTR: control; HTx: heart transplantation; NSAIDs: nonsteroidal anti-inflammatory drugs; OPCAB: off-pump coronary artery bypass grafting; POAF: postoperative atrial fibrillation; TAVR: transcatheter aortic valve replacement; SAVR: surgical aortic valve replacement.

**Table 2:** Summary of research studies on different pharmacological drugs as anti-fibrotic therapies for cardiac fibrosis through different mechanistic pathways.

Reference	Drug or Inhibitor	Animal or/and Cell Models	Induced Cardiac Disease	Main Effects of the Selected Treatment	Conclusive Remarks
Künzel et al. (2023) [151]	Mesalazine	MI mice	Fibrosis HF	- Reduced perivascular fibrosis and myocardial collagen 1A1 expression after MI. - Did not affect the total and interstitial cardiac fibrosis in MI mice.	Potential therapy against cardiac fibrosis in MI.
Silva et al. (2021) [152]	RAAS inhibitor (almandine).	LV pressure overload induced by TAC in mice.	Hypertrophy Fibrosis	- Reduced collagen deposition, CMs hyper-trophy and, the expression of cardiac remodeling-associated genes such as TGF-β and MMP-2 in the LV. - Attenuated the increase in phosphorylation of ERK1/2 and reverses the decrease in AMPKα phosphorylation in TAC mice. - Improved cardiac remodeling induced by pressure overload including oxidative stress, hypertrophy, and fibrosis.	Cardioprotective role in the treatment of cardiac diseases induced by pressure overload
Gao et al. (2021) [153]	NLRP3 inhibitor (oridonin)	MI mouse model	Fibrosis	- Improved LV ejection fraction and preserved fractional shortening. - Reduced the myocardial infarct size and fibrosis post-MI. - Reduced neutrophil and macrophage infiltration and expression levels of NLRP3, IL-1β, IL-18.	Potential therapeutic target for the treatment of acute MI.
Gao et al. (2020) [154]	TNAP inhibitor (tetramisole)	MI rat model MI patients	Fibrosis	- Improved cardiac function and decreased fibrosis post-MI. - Attenuated collagen deposition and the differentiation of myo-fibroblasts (FBs) post-MI.	New antifibrotic drug regulating cardiac fibrosis through AMPK-TGF-β1/Smads and p53 signals.
Burke et al. (2019) [155]	ARB (valsartan) and neprilysin inhibitor (sacubitril)	LV pressure overload induced by TAC in mice.	HF Fibrosis	- Reduced cardiac fibrosis and improved cardiac function. - Modulated PKG signaling in cardiac FBs and suppress Rho activation that is accompanied by myoFBs activation and proliferation.	Prevented the pressure overload induced cardiac dysfunction and fibrosis.Potential antifibrotic drugs for HFpEF.
Liu et al. (2018) [156]	H_2_S	Diabetes induced by intraperitoneal injection of STZ	Diabetic cardiomyopathyFibrosis	- Decreased the expression levels of collagen III, TGF-β, MMP8, TIMP2, caspase-3, TNF-α, NF-κB, JAK-1/2 and STAT1/3/5/6 in STZ rats. - Decreased cardiac fibrosis induced by STZ in rats.	Ameliorates cardiac fibrosis in diabetic rats by inhibiting the JAK/STAT pathway, suggesting its role as a novel therapeutic approach for diabetic cardiomyopathy.
Merino et al. (2016) [158]	BMP-7	Patients with ASPressure overload by TAC in mice Cultured CMs and FBs BMP7^+/-^mice	Hypertrophy Fibrosis	- Prevented TGF-β-induced hypertrophy in cultured CMs and transcriptional activity of collagen 1A1 in FBs - Attenuated the myocardial changes in TAC mice. - BMP-7 signaling loss in mice hinders LV reverse remodeling, but recombinant BMP-7 improves it.	The imbalance between TGF-β versus BMP-7 opposing signals may play a significant role in the induction of cardiac changes in response to hemodynamic stress.
Henry et al. (2016) [159]	Relaxin	Aged rats (24-months old)	AF Fibrosis	- Suppressed persistent AF, increased atrial CV, and reduced atrial fibrosis. - Decreased the mRNA expression of TGF-β1 and COL I and III while increasing the expression of voltage-gated sodium channel (Nav1.5) in the atria of aged rats.	Potential therapy for AF in elderly people by reversing atrial fibrosis and modifying ionic currents.
Shimada et al. (2013) [160]	ARB Losartan	Nonobstructive HCM patients	Hypertrophy Fibrosis	- Decreased LV mass and collagen volume as well as the expression of TGF-β and COL1-α.	Reduced cardiac fibrosis and hypertrophy.
Zhang et al. (2012) [161]	FT011	MI induced by left anterior descending coronary artery ligation in rats	HF Fibrosis	- Improved cardiac function and decreased collagen deposition upon FT011 treatment. - Reduced interstitial infiltration of pro- macrophages, CMs hypertrophy, and mRNA expression of COLI /III.	Limits cardiac fibrosis and improves systolic function.Potential for the treatment of cardiac fibrosis and heart failure.
Nguyen et al. (2010) [162]	TGF-β1 inhibitor Pirfenidone	MI rat model	Ventricular tachycardia Fibrosis	- Decreased VT inducibility and increased CV. - Decreased infarct dense scar area and LV fibrosis.	Reduce the infarct area and post-MI arrhythmias.

Abbreviations from Table 2. AF: atrial fibrillation; AMPKα: 5′ adenosine monophosphate-activated protein kinase; ARB: angiotensin-receptor blockers; AS: aortic stenosis; BMP-7: bone morphogenetic protein-7; CMs: cardiomyocytes; COL1: collagen type I; COLIII: collagen type III; CV: conduction velocity; ERK1/2: phospho-p44/42 MAPK extracellular signal-regulated kinases; FBs: fibroblasts; H2S: hydrogen sulfide; HCM: hypertrophic cardiomyopathy; HF: heart failure; HFpEF: heart failure with preserved ejection fraction; IL-18: interleukin (IL)-18; IL-1β: interleukin (IL)-1β; JAK1/2: Janus kinase 1/2; LV: Left ventricle; MI: myocardial infarction; MMP-2: matrix metalloproteinase-2; NF-κB: nuclear factor kappa-light-chain-enhancer of activated B; NLRP3: NACHT, LRR, and PYD domain-containing protein 3; PKG: protein kinase G; RAAS: renin-angiotensin-aldosterone system; STAT1/3/5/6: signal transducer and activator of transcription; STZ: streptozotocin; TAC: transverse aortic constriction; TGF-β: transforming growth factor bêta; TIMP2: tissue inhibitor of metalloproteinases 2. TNAP: tissue nonspecific alkaline phosphatase; TNF-α: tumor necrosis factor- α; VT: ventricular tachycardia.

## Data Availability

All original data, figures, and information are included in the paper in its original form. Additional information can be requested directly from the corresponding author: roddy.hiram@icm-mhi.org.

## References

[1] Saleh K., Haldar S. (2023). Atrial fibrillation: A contemporary update. Clin. Med..

[2] Antzelevitch C., Burashnikov A. (2011). Overview of Basic Mechanisms of Cardiac Arrhythmia. Card. Electrophysiol. Clin..

[3] Bordignon S., Corti M.C., Bilato C. (2012). Atrial Fibrillation Associated with Heart Failure, Stroke and Mortality. J. Atr. Fibrillation.

[4] Brandes A., Smit M.D., Nguyen B.O., Rienstra M., Van Gelder I.C. (2018). Risk Factor Management in Atrial Fibrillation. Arrhythmia Electrophysiol. Rev..

[5] Chung M.K., Refaat M., Shen W.-K., Kutyifa V., Cha Y.-M., Di Biase L., Baranchuk A., Lampert R., Natale A., Fisher J. (2020). Atrial Fibrillation. Circ..

[6] Olshansky B., Rosenfeld L.E., Warner A.L., Solomon A.J., O’Neill G., Sharma A., Platia E., Feld G.K., Akiyama T., Brodsky M.A. (2004). The Atrial Fibrillation Follow-up Investigation of Rhythm Management (AFFIRM) study. J. Am. Coll. Cardiol..

[7] Gutierrez C., Blanchard D.G. (2016). Diagnosis and Treatment of Atrial Fibrillation. Atr Fibrillation..

[8] Andrade J.G., Aguilar M., Atzema C., Bell A., Cairns J.A., Cheung C.C., Cox J.L., Dorian P., Gladstone D.J., Healey J.S. (2020). The 2020 Canadian Cardiovascular Society/Canadian Heart Rhythm Society Comprehensive Guidelines for the Management of Atrial Fibrillation. Can. J. Cardiol..

[9] Hindricks G., Packer D.L. (2022). Catheter ablation of atrial fibrillation: Recent advances and future challenges. Eurospace.

[10] Wasmer K., Eckardt L., Breithardt G. (2017). Predisposing factors for atrial fibrillation in the elderly. J. Geriatr. Cardiol..

[11] Čarná Z., Osmančík P. (2021). The effect of obesity, hypertension, diabetes mellitus, alcohol, and sleep apnea on the risk of atrial fibrillation. Physiol. Res..

[12] Hiram R., Provencher S. (2021). Pulmonary Disease, Pulmonary Hypertension and Atrial Fibrillation. Card. Electrophysiol. Clin..

[13] Mehdizadeh M., Naud P., Abu-Taha I.H., Hiram R., Xiong F., Xiao J., Saljic A., Kamler M., Vuong-Robillard N., Thorin E. (2024). The role of cellular senescence in profibrillatory atrial remodelling associated with cardiac pathology. Cardiovasc. Res..

[14] Peretto G., Durante A., Limite L.R., Cianflone D. (2014). Postoperative Arrhythmias after Cardiac Surgery: Incidence, Risk Factors, and Therapeutic Management. Cardiol. Res. Pr..

[15] Lowres N., Mulcahy G., Jin K., Gallagher R., Neubeck L., Freedman B. (2017). Incidence of postoperative atrial fibrillation recurrence in patients discharged in sinus rhythm after cardiac surgery: A systematic review and meta-analysis†. Interact. Cardiovasc. Thorac. Surg..

[16] Dobrev D., Aguilar M., Heijman J., Guichard J.-B., Nattel S. (2019). Postoperative atrial fibrillation: Mechanisms, manifestations and management. Nat. Rev. Cardiol..

[17] Joshi K.K., Tiru M., Chin T., Fox M.T., Stefan M.S. (2015). Postoperative atrial fibrillation in patients undergoing non-cardiac non-thoracic surgery: A practical approach for the hospitalist. Hosp. Pr..

[18] AlTurki A., Marafi M., Proietti R., Cardinale D., Blackwell R., Dorian P., Bessissow A., Vieira L., Greiss I., Essebag V. (2020). Major Adverse Cardiovascular Events Associated With Postoperative Atrial Fibrillation After Noncardiac Surgery: A System-atic Review and Meta-Analysis. Circ Arrhythm Electrophysiol..

[19] Alghosoon H., Arafat A.A., Albabtain M.A., Alsubaie F.F., Alangari A.S. (2023). Long-Term Effects of Postoperative Atrial Fibrillation following Mitral Valve Surgery. J. Cardiovasc. Dev. Dis..

[20] Mostafa A., A El-Haddad M., Shenoy M., Tuliani T. (2012). Atrial fibrillation post cardiac bypass surgery. Avicenna J. Med..

[21] Ryan T., Grindal A., Jinah R., Um K.J., Vadakken M.E., Pandey A., Jaffer I.H., Healey J.S., Belley-Coté É.P., McIntyre W.F. (2022). New-Onset Atrial Fibrillation After Transcatheter Aortic Valve Replacement: A Systematic Review and Meta-Analysis. JACC Cardiovasc. Interv..

[22] Chokesuwattanaskul R., Bathini T., Thongprayoon C., Preechawat S., O’Corragain O.A., Pachariyanon P., Ungprasert P., Cheungpasitporn W. (2018). Atrial fibrillation following heart transplantation: A systematic review and meta-analysis of observational studies. J. Evidence-Based Med..

[23] Ihara K., Sasano T. (2022). Role of Inflammation in the Pathogenesis of Atrial Fibrillation. Front. Physiol..

[24] Kota R., Gemelli M., Dimagli A., Suleiman S., Moscarelli M., Dong T., Angelini G.D., Fudulu D.P. (2023). Patterns of cytokine release and association with new onset of post-cardiac surgery atrial fibrillation. Front. Surg..

[25] Lim H.S., Schultz C., Dang J., Alasady M., Lau D.H., Brooks A.G., Wong C.X., Roberts-Thomson K.C., Young G.D., Worthley M.I. (2014). Time Course of Inflammation, Myocardial Injury, and Prothrombotic Response After Radiofrequency Catheter Ablation for Atrial Fibrillation. Circ. Arrhythmia Electrophysiol..

[26] Tao H., Shen X., Zou L., Zhang C., Hong L. (2024). Left atrial volume index and interleukin-6 as predictors for postoperative atrial fibrillation. J. Cardiothorac. Surg..

[27] Halade G.V., Lee D.H. (2022). Inflammation and resolution signaling in cardiac repair and heart failure. EBioMedicine.

[28] Younes R., LeBlanc C.-A., Hiram R. (2022). Evidence of Failed Resolution Mechanisms in Arrhythmogenic Inflammation, Fibrosis and Right Heart Disease. Biomolecules.

[29] Tisdale J.E., Wroblewski H.A., Kesler K.A. (2011). Prophylaxis of Atrial Fibrillation After Noncardiac Thoracic Surgery. Semin. Thorac. Cardiovasc. Surg..

[30] Onaitis M., D’Amico T., Zhao Y., O’Brien S., Harpole D. (2010). Risk Factors for Atrial Fibrillation After Lung Cancer Surgery: Analysis of The Society of Thoracic Surgeons General Thoracic Surgery Database. Ann. Thorac. Surg..

[31] Helgadottir S., Sigurdsson M.I., Ingvarsdottir I.L., O Arnar D., Gudbjartsson T. (2012). Atrial fibrillation following cardiac surgery: Risk analysis and long-term survival. J. Cardiothorac. Surg..

[32] Mathew J.P., Fontes M.L., Tudor I.C., Ramsay J., Duke P., Mazer C.D., Barash P.G., Hsu P.H., Mangano D.T., Foundation E. (2004). A Multicenter Risk Index for Atrial Fibrillation After Cardiac Surgery. JAMA.

[33] Lohchab S.S., Kumar A. (2020). Post-operative atrial fibrillation after off-pump coronary artery bypass grafting. Indian J. Thorac. Cardiovasc. Surg..

[34] Greenberg J.W., Lancaster T.S., Schuessler R.B., Melby S.J. (2017). Postoperative atrial fibrillation following cardiac surgery: A persistent complication. Eur. J. Cardio-Thoracic Surg..

[35] Kohno H., Ueda H., Matsuura K., Tamura Y., Watanabe M., Matsumiya G. (2017). Long-term consequences of atrial fibrillation after aortic valve replacement. Asian Cardiovasc. Thorac. Ann..

[36] Jessurun E.R., van Hemel N.M., Kelder J.C., Elbers S., de la Rivière A.B., Defauw J.J., Ernst J.M. (2000). Mitral valve surgery and atrial fibrillation: Is atrial fibrillation surgery also needed?. Eur. J. Cardio-Thoracic Surg..

[37] Vavuranakis M., Kolokathis A.-M., Vrachatis D.A., Kalogeras K., Magkoutis N.A., Fradi S., Ghostine S., Karamanou M., Tousoulis D. (2016). Atrial Fibrillation During or After TAVI: Incidence, Implications and Therapeutical Considerations. Curr. Pharm. Des..

[38] Herold J., Herold-Vlanti V., Sherif M., Luani B., Breyer C., Bonaventura K., Braun-Dullaeus R. (2017). Analysis of cardiovascular mortality, bleeding, vascular and cerebrovascular events in patients with atrial fibrillation vs. sinus rhythm undergoing transfemoral Transcatheter Aortic Valve Implantation (TAVR). BMC Cardiovasc. Disord..

[39] Smith C.R., Leon M.B., Mack M.J., Miller D.C., Moses J.W., Svensson L.G., Tuzcu E.M., Webb J.G., Fontana G.P., Makkar R.R. (2011). Transcatheter versus surgical aortic-valve replacement in high-risk patients. N. Engl. J. Med..

[40] Mack M.J., Leon M.B., Thourani V.H., Makkar R., Kodali S.K., Russo M., Kapadia S.R., Malaisrie S.C., Cohen D.J., Pibarot P. (2019). Transcatheter Aortic-Valve Replacement with a Balloon-Expandable Valve in Low-Risk Patients. N. Engl. J. Med..

[41] Mack M.J., Leon M.B., Thourani V.H., Pibarot P., Hahn R.T., Genereux P., Kodali S.K., Kapadia S.R., Cohen D.J., Pocock S.J. (2023). Transcatheter Aortic-Valve Replacement in Low-Risk Patients at Five Years. N. Engl. J. Med..

[42] Adams D.H., Popma J.J., Reardon M.J., Yakubov S.J., Coselli J.S., Deeb G.M., Gleason T.G., Buchbinder M., Hermiller J., Kleiman N.S. (2014). Transcatheter aortic-valve replacement with a self-expanding prosthesis. N. Engl. J. Med..

[43] Reardon M.J., Van Mieghem N.M., Popma J.J., Kleiman N.S., Søndergaard L., Mumtaz M., Adams D.H., Deeb G.M., Maini B., Gada H. (2017). Surgical or Transcatheter Aortic-Valve Replacement in Intermediate-Risk Patients. N. Engl. J. Med..

[44] Thyregod H.G.H., Steinbrüchel D.A., Ihlemann N., Nissen H., Kjeldsen B.J., Petursson P., Chang Y., Franzen O.W., Engstrøm T., Clemmensen P. (2015). Transcatheter Versus Surgical Aortic Valve Replacement in Patients With Severe Aortic Valve Stenosis: 1-Year Results From the All-Comers NOTION Randomized Clinical Trial. J. Am. Coll. Cardiol..

[45] Popma J.J., Deeb G.M., Yakubov S.J., Mumtaz M., Gada H., O’Hair D., Bajwa T., Heiser J.C., Merhi W., Kleiman N.S. (2019). Transcatheter Aortic-Valve Replacement with a Self-Expanding Valve in Low-Risk Patients. N. Engl. J. Med..

[46] Altaii H., Morcos R., Riad F., Abdulameer H., Khalili H., Maini B., Lieberman E., Vivas Y., Wiegn P., AJoglar J. (2020). Incidence of Early Atrial Fibrillation After Transcatheter versus Surgical Aortic Valve Replacement: A Meta-Analysis of Randomized Controlled Trials. J. Atr. Fibrillation.

[47] de Miguel I.M., Ávila P. (2021). Atrial Fibrillation in Congenital Heart Disease. Eur. Cardiol. Rev..

[48] Labombarda F., Hamilton R., Shohoudi A., Aboulhosn J., Broberg C.S., Chaix M.A., Cohen S., Cook S., Dore A., Fernandes S.M. (2017). Increasing Prevalence of Atrial Fibrillation and Permanent Atrial Arrhythmias in Congenital Heart Disease. J. Am. Coll. Cardiol..

[49] Mandalenakis Z., Rosengren A., Lappas G., Eriksson P., Gilljam T., Hansson P.-O., Skoglund K., Fedchenko M., Dellborg M. (2018). Atrial Fibrillation Burden in Young Patients With Congenital Heart Disease. Circulation.

[50] Ferro C.R.C., de Oliveira D.C., Nunes F.P., Piegas L.S. (2009). Postoperative atrial fibrillation after cardíaca. Arq. Bras. Cardiol..

[51] Bounader K., Flécher E. (2024). End-stage heart failure: The future of heart transplant and artificial heart. Presse Med..

[52] Negargar S., Sadeghi S. (2023). Early Postoperative Cardiac Complications Following Heart Transplantation. Galen Med. J..

[53] Thajudeen A., Stecker E.C., Shehata M., Patel J., Wang X., McAnulty J.J.H., Kobashigawa J., Chugh S.S. (2012). Arrhythmias After Heart Transplantation: Mechanisms and Management. J. Am. Hear. Assoc..

[54] Darche F.F., Helmschrott M., Rahm A., Thomas D., Schweizer P.A., Bruckner T., Ehlermann P., Kreusser M.M., Warnecke G., Frey N. (2021). Atrial fibrillation before heart transplantation is a risk factor for post-transplant atrial fibrillation and mortality. ESC Hear. Fail..

[55] Haïssaguerre M., Jaïs P., Shah D.C., Takahashi A., Hocini M., Quiniou G., Garrigue S., Le Mouroux A., Le Métayer P., Clémenty J. (1998). Spontaneous Initiation of Atrial Fibrillation by Ectopic Beats Originating in the Pulmonary Veins. N. Engl. J. Med..

[56] Parameswaran R., Al-Kaisey A.M., Kalman J.M. (2021). Catheter ablation for atrial fibrillation: Current indications and evolving technologies. Nat. Rev. Cardiol..

[57] Macle L., Novak P., Khairy P., Thibault B., Talajic M., Dubuc M., Roy D., Guerra P.G. (2009). Pulmonary vein isolation and left atrial catheter ablation using a three-dimensional navigation system for the treatment of atrial fibrillation. Can. J. Cardiol..

[58] Cunha P.S., Portugal G., Laranjo S., Alves M., Papoila A.L., Valente B., Delgado A.S., Lousinha A., Paulo M., Brás M. (2022). The atrial fibrillation burden during the blanking period is predictive of time to recurrence after catheter ablation. Int. J. Cardio Hear. Vasc..

[59] Chew D.S., Jones K.A., Loring Z., Black-Maier E., Noseworthy P.A., Exner D.V., Packer D.L., Grant J., Mark D.B., Piccini J.P. (2022). Diagnosis-to-ablation time predicts recurrent atrial fibrillation and rehospitalization following catheter ablation. Hear. Rhythm. O2.

[60] Andrade J.G., Champagne J., Dubuc M., Deyell M.W., Verma A., Macle L., Leong-Sit P., Novak P., Badra-Verdu M., Sapp J. (2019). Cryoballoon or Radiofrequency Ablation for Atrial Fibrillation Assessed by Continuous Monitoring: A Randomized Clinical Trial. Circulation.

[61] Freeman J.V., Tabada G.H., Reynolds K., Sung S.H., Liu T.I., Gupta N., Go A.S. (2018). Contemporary Procedural Complications, Hospitalizations, and Emergency Visits After Catheter Ablation for Atrial Fibrillation. Am. J. Cardiol..

[62] Arora S., Lahewala S., Tripathi B., Mehta V., Kumar V., Chandramohan D., Lemor A., Dave M., Patel N., Patel N.V. (2018). Causes and Predictors of Readmission in Patients With Atrial Fibrillation Undergoing Catheter Ablation: A National Population-Based Cohort Study. J. Am. Hear. Assoc. Cardiovasc. Cerebrovasc Dis..

[63] Calkins H., Brugada J., Packer D.L., Cappato R., Chen S.-A., Crijns H.J.G., Damiano R.J., Davies D.W., Haines D.E., Haissaguerre M. (2007). HRS/EHRA/ECAS Expert Consensus Statement on Catheter and Surgical Ablation of Atrial Fibrillation: Recommendations for Personnel, Policy, Procedures and Follow-UpA report of the Heart Rhythm Society (HRS) Task Force on Catheter and Surgical Ablation of Atrial Fibrillation Developed in partnership with the European Heart Rhythm Association (EHRA) and the European Cardiac Arrhythmia Society (ECAS); in collaboration with the American College of Cardiology (ACC), American Heart Association (AHA), and the Society of Thoracic Surgeons (STS). Endorsed and Approved by the governing bodies of the American College of Cardiology, the American Heart Association, the European Cardiac Arrhythmia Society, the European Heart Rhythm Association, the Society of Thoracic Surgeons, and the Heart Rhythm Society. Europace.

[64] Liang J.J., Dixit S., Santangeli P. (2016). Mechanisms and clinical significance of early recurrences of atrial arrhythmias after catheter ablation for atrial fibrillation. World J. Cardiol..

[65] Das M., Wynn G.J., Morgan M., Lodge B., Waktare J.E., Todd D.M., Hall M.C., Snowdon R.L., Modi S., Gupta D. (2015). Recurrence of Atrial Tachyarrhythmia During the Second Month of the Blanking Period Is Associated With More Extensive Pulmonary Vein Reconnection at Repeat Electrophysiology Study. Circ. Arrhythmia Electrophysiol..

[66] Liang J.J., Elafros M.A., Chik W.W., Santangeli P., Zado E.S., Frankel D.S., Supple G.E., Schaller R.D., Lin D., Hutchinson M.D. (2015). Early recurrence of atrial arrhythmias following pulmonary vein antral isolation: Timing and frequency of early recurrences predicts long-term ablation success. Hear. Rhythm..

[67] Bidar E., Bramer S., Maesen B., Maessen J.G., Schotten U. (2013). Post-operative Atrial Fibrillation—Pathophysiology, Treatment and Prevention. J. Atr. Fibrillation.

[68] El Gindy D.M.K., Solayman M.H., Khorshid R., Schaalan M.F., El Wakeel L.M. (2024). Effect of Clinical and Genetic Factors on the Development of Postoperative Atrial Fibrillation After Coronary Artery Bypass Grafting (CABG) in Egyptian Patients Receiving Beta-Blockers. Cardiovasc. Drugs Ther..

[69] Christensen M.A., Bonde A., Sillesen M. (2023). Genetic risk factors for postoperative atrial fibrillation—A nationwide genome-wide association study (GWAS). Front. Cardiovasc. Med..

[70] Gaudino M., Andreotti F., Zamparelli R., Di Castelnuovo A., Nasso G., Burzotta F., Iacoviello L., Donati M.B., Schiavello R., Maseri A. (2003). The −174G/C Interleukin-6 polymorphism influences postoperative interleukin-6 levels and postoperative atrial fibrillation. is atrial fibrillation an inflammatory complication?. Circulation.

[71] Plante I., Fournier D., Mathieu P., Daleau P. (2008). A pilot study to estimate the feasibility of assessing the relationships between polymorphisms in hKv1.5 and atrial fibrillation in patients following coronary artery bypass graft surgery. Can. J. Cardiol..

[72] Kertai M.D., Li Y.-W., Li Y.-J., Shah S.H., Kraus W.E., Fontes M.L., Stafford-Smith M., Newman M.F., Podgoreanu M.V., Mathew J.P. (2014). G Protein–Coupled Receptor Kinase 5 Gene Polymorphisms Are Associated With Postoperative Atrial Fibrillation After Coronary Artery Bypass Grafting in Patients Receiving β-Blockers. Circ. Cardiovasc. Genet..

[73] Kertai M.D., Li Y.-J., Ji Y., Qi W., Lombard F.W., Shah S.H., Kraus W.E., Stafford-Smith M., Newman M.F., Milano C.A. (2015). Genome-wide association study of new-onset atrial fibrillation after coronary artery bypass grafting surgery. Am. Heart J..

[74] Shah S., Chahil V., Battisha A., Haq S., Kalra D.K. (2024). Postoperative Atrial Fibrillation: A Review. Biomedicines..

[75] Gaudino M., Di Franco A., Rong L.Q., Piccini J., Mack M. (2023). Postoperative atrial fibrillation: From mechanisms to treatment. Eur. Hear. J..

[76] Meerman M., Buijser M., Berg L.v.D., Heuvel A.-M.v.D., Hoohenkerk G., van Driel V., Munsterman L., de Vroege R., Bailey M., Bellomo R. (2024). Magnesium sulphate to prevent perioperative atrial fibrillation in cardiac surgery: A randomized clinical trial. Trials.

[77] Lopes L.A., Agrawal D.K. (2022). Post-Operative Atrial Fibrillation: Current Treatments and Etiologies for a Persistent Surgical Complication. J. Surg. Res..

[78] Howitt S.H., Grant S.W., Campbell N.G., Malagon I., McCollum C. (2020). Are Serum Potassium and Magnesium Levels Associated with Atrial Fibrillation After Cardiac Surgery?. J. Cardiothorac. Vasc. Anesthesia.

[79] E Fakuade F., Steckmeister V., Seibertz F., Gronwald J., Kestel S., Menzel J., Pronto J.R.D., Taha K., Haghighi F., Kensah G. (2021). Altered atrial cytosolic calcium handling contributes to the development of postoperative atrial fibrillation. Cardiovasc. Res..

[80] Qian S.S., Crandell I., Hanlon A., Joseph M., Poelzing S. (2022). Predictive Capability of Metabolic Panels for Postoperative Atrial Fibrillation in Cardiac Surgery Patients. J. Surg. Res..

[81] Olesen O.J., Fosbøl E.L. (2021). Sympathetic hyperactivity after coronary artery bypass graft surgery: An important player in the development of postoperative atrial fibrillation? Authors’ reply. Eurospace.

[82] Hiram R. (2021). Cardiac cytokine therapy? Relevance of targeting inflammatory mediators to combat cardiac arrhythmogenic remodeling. IJC Hear. Vasc..

[83] Jeong E.-M., Liu M., Sturdy M., Gao G., Varghese S.T., Sovari A.A., Dudley S.C. (2012). Metabolic stress, reactive oxygen species, and arrhythmia. J. Mol. Cell. Cardiol..

[84] Harada M., Nattel S. (2021). Implications of Inflammation and Fibrosis in Atrial Fibrillation Pathophysiology. Card. Electrophysiol. Clin..

[85] Odeh A., Dungan G.D., Hoppensteadt D., Siddiqui F., Kantarcioglu B., Darki A., Fareed J., Syed M.A. (2023). Interrelationship Between Inflammatory Biomarkers and Collagen Remodeling Proteins in Atrial Fibrillation. Clin. Appl. Thromb..

[86] Burstein B., Nattel S. (2008). Atrial Fibrosis: Mechanisms and Clinical Relevance in Atrial Fibrillation. J. Am. Coll. Cardiol..

[87] Rosenberg M.A., Maziarz M., Tan A.Y., Glazer N.L., Zieman S.J., Kizer J.R., Ix J.H., Djousse L., Siscovick D.S., Heckbert S.R. (2014). Circulating fibrosis biomarkers and risk of atrial fibrillation: The Cardiovascular Health Study (CHS). Am. Hear. J..

[88] Yue L., Xie J., Nattel S. (2011). Molecular determinants of cardiac fibroblast electrical function and therapeutic implications for atrial fibrillation. Cardiovasc. Res..

[89] Frustaci A., Chimenti C., Bellocci F., Morgante E., Russo M.A., Maseri A. (1997). Histological substrate of atrial biopsies in patients with lone atrial fibrillation. Circulation.

[90] Boldt A., Wetzel U., Lauschke J., Weigl J., Gummert J., Hindricks G., Kottkamp H., Dhein S. (2004). Fibrosis in left atrial tissue of patients with atrial fibrillation with and without underlying mitral valve disease. Heart.

[91] Zakkar M., Ascione R., James A., Angelini G., Suleiman M. (2015). Inflammation, oxidative stress and postoperative atrial fibrillation in cardiac surgery. Pharmacol. Ther..

[92] Castillo R.L., Farías J., Sandoval C., González-Candia A., Figueroa E., Quezada M., Cruz G., Llanos P., Jorquera G., Kostin S. (2024). Role of NLRP3 Inflammasome in Heart Failure Patients Undergoing Cardiac Surgery as a Potential Determinant of Postoperative Atrial Fibrillation and Remodeling: Is SGLT2 Cotransporter Inhibition an Alternative for Cardioprotection?. Antioxidants.

[93] Toro-Pérez J., Rodrigo R. (2021). Contribution of oxidative stress in the mechanisms of postoperative complications and multiple organ dysfunction syndrome. Redox Rep..

[94] Kramer P.A., Chacko B.K., Ravi S., Johnson M.S., Mitchell T., Barnes S., Arabshahi A., Dell’Italia L.J., George D.J., Steele C. (2014). Hemoglobin-associated oxidative stress in the pericardial compartment of postoperative cardiac surgery patients. Mod. Pathol..

[95] Worden J.C., Asare K. (2014). Postoperative atrial fibrillation: Role of inflammatory biomarkers and use of colchicine for its prevention. Pharmacother. J. Hum. Pharmacol. Drug Ther..

[96] Abdelhadi R.H., Gurm H.S., Van Wagoner D.R., Chung M.K. (2004). Relation of an exaggerated rise in white blood cells after coronary bypass or cardiac valve surgery to development of atrial fibrillation postoperatively. Am. J. Cardiol..

[97] Sabol F., Jakubová M., Mitro P., Bomba A., Chmelárová A., Petrášová D., Stančák B., Nagy V., Török P., ebová S. (2012). Is there a relationship between inflammatory markers, oxidative stress and postopera-tive atrial fibrillation?. Vnitr Lek..

[98] Fontes M.L., Mathew J.P., Rinder H.M., Zelterman D., Smith B.R., Rinder C.S. (2005). Atrial Fibrillation After Cardiac Surgery/Cardiopulmonary Bypass Is Associated with Monocyte Activation. Anesthesia Analg..

[99] Gibson P.H., Cuthbertson B.H., Croal B.L., Rae D., El-Shafei H., Gibson G., Jeffrey R.R., Buchan K.G., Hillis G.S. (2010). Usefulness of Neutrophil/Lymphocyte Ratio As Predictor of New-Onset Atrial Fibrillation After Coronary Artery Bypass Grafting. Am. J. Cardiol..

[100] Hoffman B.F., Feinmark S.J., Guo S. (1997). Electrophysiologic Effects of Interactions Between Activated Canine Neutrophils and Cardiac Myocytes. J. Cardiovasc. Electrophysiol..

[101] Grune J., Lewis A.J.M., Yamazoe M., Hulsmans M., Rohde D., Xiao L., Zhang S., Ott C., Calcagno D.M., Zhou Y. (2022). Neutrophils incite and macrophages avert electrical storm after myocardial infarction. Nat. Cardiovasc. Res..

[102] Yao C., Veleva T., Scott L., Cao S., Li L., Chen G., Jeyabal P., Pan X., Alsina K.M., Abu-Taha I. (2018). Enhanced Cardiomyocyte NLRP3 Inflammasome Signaling Promotes Atrial Fibrillation. Circulation.

[103] Sundaram D.M., Vasavada A.M., Ravindra C., Rengan V., Sundaram P.M. (2023). The Management of Postoperative Atrial Fibrillation (POAF): A Systematic Review. Cureus.

[104] Suero O.R., Ali A.K., Barron L.R., Segar M.W., Moon M.R., Chatterjee S. (2024). Postoperative atrial fibrillation (POAF) after cardiac surgery: Clinical practice review. J. Thorac. Dis..

[105] Ahmed M., Belley-Coté E.P., Qiu Y., Belesiotis P., Tao B., Wolf A., Kaur H., Ibrahim A., Wong J.A., Wang M.K. (2023). Rhythm vs. Rate Control in Patients with Postoperative Atrial Fibrillation after Cardiac Surgery: A Systematic Review and Meta-Analysis. J. Clin. Med..

[106] Fauchier L., Laborie G., Clementy N., Babuty D. (2016). Beta-blockers or Digoxin for Atrial Fibrillation and Heart Failure?. Card. Fail. Rev..

[107] Turagam M.K., Downey F.X., Kress D.C., Sra J., Tajik A.J., Jahangir A. (2015). Pharmacological strategies for prevention of postoperative atrial fibrillation. Expert Rev. Clin. Pharmacol..

[108] Beukema R.J., Adiyaman A., Smit J.J.J., Delnoy P.P.H., Misier A.R.R., Elvan A. (2016). Catheter ablation of symptomatic postoperative atrial arrhythmias after epicardial surgical disconnection of the pulmonary veins and left atrial appendage ligation in patients with atrial fibrillation. Eur. J. Cardio-Thoracic Surg..

[109] Hindricks G., Potpara T., Dagres N., Arbelo E., Bax J.J., Blomström-Lundqvist C., Boriani G., Castella M., Dan G.A., Dilaveris P.E. (2021). 2020 ESC Guidelines for the diagnosis and management of atrial fibrillation developed in collaboration with the European Association for Cardio-Thoracic Surgery (EACTS): The Task Force for the diagnosis and management of atrial fibrillation of the European Society of Cardiology (ESC) Developed with the special contribution of the European Heart Rhythm Association (EHRA) of the ESC. Eur. Heart J..

[110] Joglar J.A., Chung M.K., Armbruster A.L., Benjamin E.J., Chyou J.Y., Cronin E.M., Deswal A., Eckhardt L.L., Goldberger Z.D., Gopinathannair R. (2024). 2023 ACC/AHA/ACCP/HRS Guideline for the Diagnosis and Management of Atrial Fibrillation: A Report of the American College of Cardiology/American Heart Association Joint Committee on Clinical Practice Guidelines. Circulation.

[111] Ishii Y., Schuessler R.B., Gaynor S.L., Hames K., Damiano R.J. (2017). Postoperative atrial fibrillation: The role of the inflammatory response. J. Thorac. Cardiovasc. Surg..

[112] Liu D., Li Y., Zhao Q. (2023). Effects of Inflammatory Cell Death Caused by Catheter Ablation on Atrial Fibrillation. J. Inflamm. Res..

[113] Anselmi A., Possati G., Gaudino M. (2009). Postoperative inflammatory reaction and atrial fibrillation: Simple correlation or causation?. Ann. Thorac. Surg..

[114] Nomani H., Mohammadpour A.H., Moallem S.M.H., Sahebkar A. (2019). Anti-inflammatory drugs in the prevention of post-operative atrial fibrillation: A literature review. Inflammopharmacology.

[115] Robinson P.C., Terkeltaub R., Pillinger M.H., Shah B., Karalis V., Karatza E., Liew D., Imazio M., Cornel J.H., Thompson P.L. (2022). Consensus Statement Regarding the Efficacy and Safety of Long-Term Low-Dose Colchicine in Gout and Cardiovascular Disease. Am. J. Med..

[116] Buckley L.F., Libby P. (2024). Colchicine’s Role in Cardiovascular Disease Management. Arter. Thromb. Vasc. Biol..

[117] Alunno A., Carubbi F., Ferri C. (2024). Colchicine and cardiovascular prevention. Eur. J. Intern. Med..

[118] Dalbeth N., Lauterio T.J., Wolfe H.R. (2014). Mechanism of action of colchicine in the treatment of gout. Clin. Ther..

[119] Silvis M.J., Fiolet A.T., Opstal T.S., Dekker M., Suquilanda D., Zivkovic M., Duyvendak M., The S.H., Timmers L., Bax W.A. (2021). Colchicine reduces extracellular vesicle NLRP3 inflammasome protein levels in chronic coronary disease: A LoDoCo2 biomarker substudy. Atherosclerosis.

[120] Ma Z., Chen J., Jin K., Chen X. (2022). Colchicine and coronary heart disease risks: A meta-analysis of randomized controlled clinical trials. Front. Cardiovasc. Med..

[121] Wu Q., Liu H., Liao J., Zhao N., Tse G., Han B., Chen L., Huang Z., Du Y. (2020). Colchicine prevents atrial fibrillation promotion by inhibiting IL-1β-induced IL-6 release and atrial fibrosis in the rat sterile pericarditis model. Biomed. Pharmacother..

[122] Ge P., Fu Y., Su Q., Jin M., Guo L., Miao C., Zhu S., Zhuang J., Zhang Z., Hong J. (2022). Colchicine for prevention of post-operative atrial fibrillation: Meta-analysis of randomized controlled trials. Front. Cardiovasc. Med..

[123] Imazio M., Brucato A., Ferrazzi P., Rovere M.E., Gandino A., Cemin R., Ferrua S., Belli R., Maestroni S., Simon C. (2011). Colchicine Reduces Postoperative Atrial Fibrillation. Circulation.

[124] Deftereos S., Giannopoulos G., Kossyvakis C., Efremidis M., Panagopoulou V., Kaoukis A., Raisakis K., Bouras G., Angelidis C., Theodorakis A. (2012). Colchicine for Prevention of Early Atrial Fibrillation Recurrence After Pulmonary Vein Isolation: A Randomized Controlled Study. J. Am. Coll. Cardiol..

[125] Lee J.Z., Singh N., Howe C.L., Low S.-W., Huang J.J., Ortega G., Lee K.S., Pandit A. (2016). Colchicine for Prevention of Post-Operative Atrial Fibrillation: A Meta-Analysis. JACC: Clin. Electrophysiol..

[126] Tucker B., Goonetilleke N., Patel S., Keech A. (2024). Colchicine in atherosclerotic cardiovascular disease. Heart.

[127] Nidorf S.M., Ben-Chetrit E., Ridker P.M. (2024). Low-dose colchicine for atherosclerosis: Long-term safety. Eur. Hear. J..

[128] Katkenov N., Mukhatayev Z., Kozhakhmetov S., Sailybayeva A., Bekbossynova M., Kushugulova A. (2024). Systematic Review on the Role of IL-6 and IL-1β in Cardiovascular Diseases. J. Cardiovasc. Dev. Dis..

[129] Marcus G.M., Whooley M.A., Glidden D.V., Pawlikowska L., Zaroff J.G., Olgin J.E. (2008). Interleukin-6 and atrial fibrillation in patients with coronary artery disease: Data from the Heart and Soul Study. Am. Hear. J..

[130] Li X., Wu X., Chen X., Peng S., Chen S., Zhou G., Wei Y., Lu X., Zhou C., Ye Y. (2023). Selective blockade of interleukin 6 trans-signaling depresses atrial fibrillation. Hear. Rhythm..

[131] Kow C.S., Ramachandram D.S., Hasan S.S. (2023). Preventing atrial fibrillation in COVID-19: Exploring the role of interleukin-6 receptor antagonists. Expert Rev. Cardiovasc. Ther..

[132] Shankar-Hari M., Vale C.L., Godolphin P.J., Fisher D., Higgins J.P.T., Spiga F., Savović J., Tierney J., Baron G., The WHO Rapid Evidence Appraisal for COVID-19 Therapies (REACT) Working Group (2021). Association Between Administration of IL-6 Antagonists and Mortality Among Patients Hospitalized for COVID-19: A Meta-analysis. JAMA.

[133] Gupta S., Wang W., Hayek S.S., Chan L., Mathews K.S., Melamed M.L., Brenner S.K., Schenck E.J., Radbel J., Reiser J. (2021). Association Between Early Treatment With Tocilizumab and Mortality Among Critically Ill Patients With COVID-19. JAMA Intern. Med..

[134] Li G., Chen Z., Bhat O.M., Zhang Q., Abais-Battad J.M., Conley S.M., Ritter J.K., Li P.-L. (2017). NLRP3 inflammasome as a novel target for docosahexaenoic acid metabolites to abrogate glomerular injury. J. Lipid Res..

[135] Hiram R., Xiong F., Naud P., Xiao J., Sosnowski D.K., Le Quilliec E., Saljic A., Abu-Taha I.H., Kamler M., LeBlanc C.-A. (2024). An inflammation resolution–promoting intervention prevents atrial fibrillation caused by left ventricular dysfunction. Cardiovasc. Res..

[136] Ghlichloo I., Gerriets V. (2025). Nonsteroidal Anti-Inflammatory Drugs (NSAIDs). StatPearls.

[137] Ong C., Lirk P., Tan C., Seymour R. (2007). An evidence-based update on nonsteroidal anti-inflammatory drugs. Clin. Med. Res..

[138] Hynninen M.S., Cheng D.C.H., Hossain I., Carroll J., Aumbhagavan S.S., Yue R., Karski J.M. (2000). Non-steroidal anti-inflammatory drugs in treatment of postoperative pain after cardiac surgery. Can. J. Anaesth..

[139] Cheruku K.K., Ghani A., Ahmad F., Pappas P., Silverman P.R., Zelinger A., Silver M.A. (2004). Efficacy of Nonsteroidal Anti-Inflammatory Medications for Prevention of Atrial Fibrillation Following Coronary Artery Bypass Graft Surgery. Prev. Cardiol..

[140] Malektojari A., Javidfar Z., Ghazizadeh S., Lahuti S., Shokraei R., Zeinaee M., Badele A., Mirzadeh R., Ashrafi M., Afra F. (2024). Effectiveness of Anti-Inflammatory Agents to Prevent Atrial Fibrillation After Cardiac Surgery: A Systematic Review and Network Meta-Analysis. CJC Open.

[141] Schmidt M., Christiansen C.F., Mehnert F., Rothman K.J., Sørensen H.T. (2011). Non-steroidal anti-inflammatory drug use and risk of atrial fibrillation or flutter: Population based case-control study. BMJ.

[142] Krijthe B.P., Heeringa J., Hofman A., Franco O.H., Stricker B.H. (2014). Non-steroidal anti-inflammatory drugs and the risk of atrial fibrillation: A population-based follow-up study. BMJ Open.

[143] De Caterina R., Ruigómez A., Rodríguez L.A.G. (2010). Long-term Use of Anti-inflammatory Drugs and Risk of Atrial Fibrillation. Arch. Intern. Med..

[144] Karamnov S., Muehlschlegel J.D. (2022). Inflammatory Responses to Surgery and Postoperative Atrial Fibrillation. Anesthesiology.

[145] Huang L., Wang C.-F., Serhan C.N., Strichartz G. (2011). Enduring prevention and transient reduction of postoperative pain by intrathecal resolvin D1. Pain.

[146] Kain V., Ingle K.A., Colas R.A., Dalli J., Prabhu S.D., Serhan C.N., Joshi M.D., Halade G.V. (2015). Resolvin D1 activates the inflammation resolving response at splenic and ventricular site following myocardial infarction leading to improved ventricular function. J. Mol. Cell. Cardiol..

[147] Xu Z.-Z., Zhang L., Liu T., Park J.Y., Berta T., Yang R., Serhan C.N., Ji R.-R. (2010). Resolvins RvE1 and RvD1 attenuate inflammatory pain via central and peripheral actions. Nat. Med..

[148] Serhan C.N. (2014). Pro-resolving lipid mediators are leads for resolution physiology. Nature.

[149] Halade G.V., Kain V., Serhan C.N. (2018). Immune responsive resolvin D1 programs myocardial infarction-induced cardiorenal syndrome in heart failure. FASEB J. Off Publ. Fed. Am. Soc. Exp. Biol..

[150] Hiram R., Naud P., Xiong F., Al-U’datt D., Algalarrondo V., Sirois M.G., Tanguay J.-F., Tardif J.-C., Nattel S. (2019). Right Atrial Mechanisms of Atrial Fibrillation in a Rat Model of Right Heart Disease. J. Am. Coll. Cardiol..

[151] Künzel S.R., Winter L., Hoffmann M., Kant T.A., Thiel J., Kronstein-Wiedemann R., Klapproth E., Lorenz K., El-Armouche A., Kämmerer S. (2023). Investigation of mesalazine as an antifibrotic drug following myocardial infarction in male mice. Physiol. Rep..

[152] Silva M.M., de Souza-Neto F.P., de Jesus I.C.G., Gonçalves G.K., Santuchi M.d.C., Sanches B.d.L., de Alcântara-Leonídio T.C., Melo M.B., Vieira M.A.R., Guatimosim S. (2021). Alamandine improves cardiac remodeling induced by transverse aortic constriction in mice. Am. J. Physiol. Circ. Physiol..

[153] Gao R.-F., Li X., Xiang H.-Y., Yang H., Lv C.-Y., Sun X.-L., Chen H.-Z., Gao Y., Yang J.-S., Luo W. (2021). The covalent NLRP3-inflammasome inhibitor Oridonin relieves myocardial infarction induced myocardial fibrosis and cardiac remodeling in mice. Int. Immunopharmacol..

[154] Gao L., Wang L.-Y., Liu Z.-Q., Jiang D., Wu S.-Y., Guo Y.-Q., Tao H.-M., Sun M., You L.-N., Qin S. (2020). TNAP inhibition attenuates cardiac fibrosis induced by myocardial infarction through deactivating TGF-β1/Smads and activating P53 signaling pathways. Cell Death Dis..

[155] Burke R.M., Lighthouse J.K., Mickelsen D.M., Small E.M. (2019). Sacubitril/Valsartan Decreases Cardiac Fibrosis in Left Ventricle Pressure Overload by Restoring PKG Signaling in Cardiac Fibroblasts. Circ. Hear. Fail..

[156] Liu M., Li Y., Liang B., Li Z., Jiang Z., Chu C., Yang J. (2018). Hydrogen sulfide attenuates myocardial fibrosis in diabetic rats through the JAK/STAT signaling pathway. Int. J. Mol. Med..

[157] Skoumal R., Tóth M., Serpi R., Rysä J., Leskinen H., Ulvila J., Saiho T., Aro J., Ruskoaho H., Szokodi I. (2011). Parthenolide inhibits STAT3 signaling and attenuates angiotensin II-induced left ventricular hypertrophy via modulation of fibroblast activity. J. Mol. Cell. Cardiol..

[158] Merino D., Villar A.V., García R., Tramullas M., Ruiz L., Ribas C., Cabezudo S., Nistal J.F., Hurlé M.A. (2016). BMP-7 attenuates left ventricular remodelling under pressure overload and facilitates reverse remodelling and functional recovery. Cardiovasc. Res..

[159] Henry B.L., Gabris B., Li Q., Martin B., Giannini M., Parikh A., Patel D., Haney J., Schwartzman D.S., Shroff S.G. (2016). Relaxin suppresses atrial fibrillation in aged rats by reversing fibrosis and upregulating Na^+^ channels. Hear. Rhythm..

[160] Shimada Y.J., Passeri J.J., Baggish A.L., O’Callaghan C., Lowry P.A., Yannekis G., Abbara S., Ghoshhajra B.B., Rothman R.D., Ho C.Y. (2013). Effects of Losartan on Left Ventricular Hypertrophy and Fibrosis in Patients With Nonobstructive Hypertrophic Cardiomyopathy. JACC Heart Fail..

[161] Zhang Y., Elsik M., Edgley A.J., Cox A.J., Kompa A.R., Wang B., Tan C.Y.R., Khong F.L., Stapleton D.I., Zammit S. (2012). A new anti-fibrotic drug attenuates cardiac remodeling and systolic dysfunction following experimental myocardial infarction. Int. J. Cardiol..

[162] Nguyen D.T., Ding C., Wilson E., Marcus G.M., Olgin J.E. (2010). Pirfenidone mitigates left ventricular fibrosis and dysfunction after myocardial infarction and reduces arrhythmias. Hear. Rhythm..

[163] Al-U’Datt D.G., Tranchant C.C., Al-Dwairi A., Alqudah M., Al-Shboul O., Hiram R., Allen B.G., Jaradat S., Alqbelat J., Abu-Zaiton A.S. (2022). Implications of enigmatic transglutaminase 2 (TG2) in cardiac diseases and therapeutic developments. Biochem. Pharmacol..

[164] Gaudino M., Di Franco A., Rong L.Q., Cao D., Pivato C.A., Soletti G.J., Chadow D., Cancelli G., Olaria R.P., Gillinov M. (2022). Pericardial Effusion Provoking Atrial Fibrillation After Cardiac Surgery: JACC Review Topic of the Week. J. Am. Coll. Cardiol..

[165] Gaudino M., Sanna T., Ballman K.V., Robinson N.B., Hameed I., Audisio K., Rahouma M., Di Franco A., Soletti G.J., Lau C. (2021). Posterior left pericardiotomy for the prevention of atrial fibrillation after cardiac surgery: An adaptive, single-centre, single-blind, randomised, controlled trial. Lancet.

[166] Rabelo L.G., Zindovic I., Astrom D.O., Thorsteinsson E.G., Sjogren J., Olafsdottir K.L., Magnusdottir M.M., Jeppsson A., Gudbjartsson T. (2024). A posterior pericardial chest tube is associated with reduced incidence of postoperative atrial fibrillation after cardiac surgery: A propensity score–matched study. JTCVS Open.

[167] Turagam M.K., Mirza M., Werner P.H., Sra J., Kress D.C., Tajik A.J., Jahangir A. (2016). Circulating Biomarkers Predictive of Postoperative Atrial Fibrillation. Cardiol. Rev..

[168] Hassanabad A.F., Deniset J.F., Fedak P.W. (2023). Pericardial Inflammatory Mediators That Can Drive Postoperative Atrial Fibrillation in Cardiac Surgery Patients. Can. J. Cardiol..

[169] Lafuse W.P., Wozniak D.J., Rajaram M.V.S. (2020). Role of Cardiac Macrophages on Cardiac Inflammation, Fibrosis and Tissue Repair. Cells.

[170] Hassanabad A.F., Schoettler F.I., Kent W.D., Adams C.A., Holloway D.D., Ali I.S., Novick R.J., Ahsan M.R., McClure R.S., Shanmugam G. (2022). Comprehensive characterization of the postoperative pericardial inflammatory response: Potential implications for clinical outcomes. JTCVS Open.

[171] Heijman J., Muna A.P., Veleva T., Molina C.E., Sutanto H., Tekook M., Wang Q., Abu-Taha I.H., Gorka M., Künzel S. (2020). Atrial Myocyte NLRP3/CaMKII Nexus Forms a Substrate for Postoperative Atrial Fibrillation. Circ. Res..

[172] Pang Q., You L., Meng X., Li Y., Deng T., Li D., Zhu B. (2023). Regulation of the JAK/STAT signaling pathway: The promising targets for cardiovascular disease. Biochem. Pharmacol..

[173] Andelova K., Bacova B.S., Sykora M., Hlivak P., Barancik M., Tribulova N. (2022). Mechanisms Underlying Antiarrhythmic Properties of Cardioprotective Agents Impacting Inflammation and Oxidative Stress. Int. J. Mol. Sci..

[174] Bi X., Zhang S., Jiang H., Ma W., Li Y., Lu W., Yang F., Wei Z. (2022). Mechanistic Insights Into Inflammation-Induced Arrhythmias: A Simulation Study. Front. Physiol..

[175] Rezaei Y., Peighambari M.M., Naghshbandi S., Samiei N., Ghavidel A.A., Dehghani M.R., Haghjoo M., Hosseini S. (2020). Postoperative Atrial Fibrillation Following Cardiac Surgery: From Pathogenesis to Potential Therapies. Am. J. Cardiovasc. Drugs.

[176] Liu C., Wang J., Yiu D., Liu K. (2014). The efficacy of glucocorticoids for the prevention of atrial fibrillation, or length of intensive care unite or hospital stay after cardiac surgery: A meta-analysis. Cardiovasc. Ther..

[177] Carroll Z., Ridgway E., Owen P., Bonetti A., Ikonomidis J., Rosenkrans D., Caranasos T., McLean D., Li Q., Zhuo S. (2024). Prophylaxis for postoperative atrial fibrillation: Impact of the implementation of a medication bundle protocol. JTCVS Open.

[178] Ebrahimi F., Hirt J., Schönenberger C., Ewald H., Briel M., Janiaud P., Hemkens L.G. (2023). Colchicine for the secondary prevention of cardiovascular events. Cochrane Database Syst. Rev..

[179] Forbes W.L., Petway J., Gressler L., Thorfinnson H., Costantino R.C., Atkinson T.J. (2024). Identifying Risk Factors for Cardiovascular Events Among Active-Duty Service Members and Veterans Prescribed Nonsteroidal Anti-Inflammatory Drugs (NSAIDs). J. Pain Res..

[180] Horbach S.J., Lopes R.D., Guaragna J.C.d.C., Martini F., Mehta R.H., Petracco J.B., Bodanese L.C., Filho A.C., Cirenza C., de Paola A.A. (2011). Naproxen as prophylaxis against atrial fibrillation after cardiac surgery: The NAFARM randomized trial. Am. J. Med..

